# Prospective multicentre accuracy evaluation of the FUJIFILM SILVAMP TB LAM test for the diagnosis of tuberculosis in people living with HIV demonstrates lot-to-lot variability

**DOI:** 10.1371/journal.pone.0303846

**Published:** 2024-05-31

**Authors:** Rita Székely, Bianca Sossen, Madalo Mukoka, Monde Muyoyeta, Elizabeth Nakabugo, Jerry Hella, Hung Van Nguyen, Sasiwimol Ubolyam, Kinuyo Chikamatsu, Aurélien Macé, Marcia Vermeulen, Chad M. Centner, Sarah Nyangu, Nsala Sanjase, Mohamed Sasamalo, Huong Thi Dinh, The Anh Ngo, Weerawat Manosuthi, Supunnee Jirajariyavej, Satoshi Mitarai, Nhung Viet Nguyen, Anchalee Avihingsanon, Klaus Reither, Lydia Nakiyingi, Andrew D. Kerkhoff, Peter MacPherson, Graeme Meintjes, Claudia M. Denkinger, Morten Ruhwald, FujiLAM Study Consortium

**Affiliations:** 1 FIND, The Global Alliance for Diagnostics, Geneva, Switzerland; 2 Department of Medicine, Faculty of Health Sciences, University of Cape Town, Cape Town, South Africa; 3 Wellcome Centre for Infectious Diseases Research in Africa, Institute of Infectious Disease and Molecular Medicine, University of Cape Town, Cape Town, South Africa; 4 Public Health Group, Malawi-Liverpool-Wellcome Programme, Blantyre, Malawi; 5 Department of Pathology, Kamuzu University of Health Sciences, Blantyre, Malawi; 6 Centre for Infectious Disease Research in Zambia, Lusaka, Zambia; 7 Infectious Diseases Institute, Makerere University, Kampala, Uganda; 8 Ifakara Health Institute, Dar es Salaam, Tanzania; 9 National Lung Hospital, Ha Noi, Viet Nam; 10 HIV-NAT, Thai Red Cross AIDS Research Centre and Centre of Excellence in Tuberculosis, Faculty of Medicine, Chulalongkorn University, Bangkok, Thailand; 11 Department of Mycobacterium Reference and Research, Research Institute of Tuberculosis, Japan Anti-Tuberculosis Association, Tokyo, Japan; 12 Division of Medical Microbiology, University of Cape Town and National Health Laboratory Service, Groote Schuur Hospital, Cape Town, South Africa; 13 Viet Tiep Hospital, Hai Phong, Viet Nam; 14 Bamrasnaradura Infectious Diseases Institute, Nonthaburi, Thailand; 15 Taksin Hospital, Bangkok, Thailand; 16 Swiss Tropical and Public Health Institute, Allschwil, Switzerland; 17 University of Basel, Basel, Switzerland; 18 Division of HIV, Infectious Diseases and Global Medicine, Zuckerberg San Francisco General Hospital and Trauma Center, University of California San Francisco, San Francisco, CA, United States of America; 19 Department of Clinical Sciences, Liverpool School of Tropical Medicine, Liverpool, United Kingdom; 20 Clinical Research Department, London School of Hygiene & Tropical Medicine, London, United Kingdom; 21 Division of Infectious Disease and Tropical Medicine, Heidelberg University Hospital and Faculty of Medicine, Heidelberg University, Heidelberg, Germany; 22 German Centre for Infection Research (DZIF), Partner site Heidelberg University Hospital, Heidelberg, Germany; Burnet Institute, AUSTRALIA

## Abstract

There is an urgent need for rapid, non-sputum point-of-care diagnostics to detect tuberculosis. This prospective trial in seven high tuberculosis burden countries evaluated the diagnostic accuracy of the point-of-care urine-based lipoarabinomannan assay FUJIFILM SILVAMP TB LAM (FujiLAM) among inpatients and outpatients living with HIV. Diagnostic performance of FujiLAM was assessed against a mycobacterial reference standard (sputum culture, blood culture, and Xpert Ultra from urine and sputum at enrollment, and additional sputum culture ≤7 days from enrollment), an extended mycobacterial reference standard (eMRS), and a composite reference standard including clinical evaluation. Of 1637 participants considered for the analysis, 296 (18%) were tuberculosis positive by eMRS. Median age was 40 years, median CD4 cell count was 369 cells/ul, and 52% were female. Overall FujiLAM sensitivity was 54·4% (95% CI: 48·7–60·0), overall specificity was 85·2% (83·2–87·0) against eMRS. Sensitivity and specificity estimates varied between sites, ranging from 26·5% (95% CI: 17·4%–38·0%) to 73·2% (60·4%–83·0%), and 75·0 (65·0%–82·9%) to 96·5 (92·1%–98·5%), respectively. Post-hoc exploratory analysis identified significant variability in the performance of the six FujiLAM lots used in this study. Lot variability limited interpretation of FujiLAM test performance. Although results with the current version of FujiLAM are too variable for clinical decision-making, the lipoarabinomannan biomarker still holds promise for tuberculosis diagnostics. The trial is registered at clinicaltrials.gov (NCT04089423).

## Introduction

Tuberculosis (TB) is a leading cause of death from a single infectious disease, second only recently to COVID-19 [1]. In 2021, TB caused 1·6 million deaths, including 187,000 among people living with HIV (PLHIV) [2]. TB is the most common cause of death in PLHIV, who have a 30-times greater risk of developing TB disease than those without HIV [3]. Most of the deaths from TB would be preventable if TB were diagnosed earlier, yet TB often goes undiagnosed [4–6].

Traditional diagnostic methods for TB, such as culture or smear microscopy, are slow or have low sensitivity. More sensitive modern techniques, such as Xpert^®^ MTB/RIF (Cepheid, Sunnyvale, CA, USA), require some laboratory infrastructure, are costly, and often inaccessible at the primary healthcare level where people at greatest risk of TB disease are more likely to seek care. Moreover, TB is harder to diagnose in PLHIV, who frequently have paucibacillary, extrapulmonary or disseminated TB, and often experience difficulty producing sputum specimens [7, 8]. TB in PLHIV is associated with high mortality if undiagnosed or if treatment is delayed [9]. New, rapid, non-sputum-based point-of-care (POC) diagnostic tests to detect TB are urgently needed [10].

FUJIFILM SILVAMP TB LAM (FujiLAM; Fujifilm, Tokyo, Japan) is a visually read, qualitative, rapid, in vitro diagnostic test for the detection of the lipoarabinomannan (LAM) antigen of *Mycobacterium tuberculosis* (MTB) in human urine [11]. FujiLAM includes two monoclonal antibodies, which bind to glycan capping motifs of LAM, and a silver amplification immunochromatography step, which enables an approximately 30-fold lower limit of detection compared with conventional lateral flow immunoassays (e.g. the Determine™ TB LAM Ag “AlereLAM”, Abbott, Chicago, IL, USA) [11, 12]. The binding targets of glycan capping motifs also result in increased specificity for MTB complex [13].

In a study of frozen urine samples from inpatient PLHIV, FujiLAM showed superior diagnostic sensitivity (70% vs. 42%) with similar specificity (91% vs. 95%) to AlereLAM [11, 14]. Here, we report results from a large-scale, multicentre evaluation of FujiLAM accuracy on prospectively collected, fresh urine samples from PLHIV against a comprehensive reference standard.

## Methods

### Study design and participants

This was a prospective, multicentre cohort study, with consecutive patient recruitment from clinical sites in seven high TB burden countries (Malawi, South Africa, Tanzania, Thailand, Uganda, Viet Nam and Zambia), between December 2019 and July 2021. Participant follow-up was completed in February 2022. Participating centres are described in Table 1 in S5 File. The study recruited adult (≥18 years) PLHIV, irrespective of CD4 counts and antiretroviral therapy (ART) status, who had received no or <3 doses of anti-TB treatment in the last 60 days and no isoniazid preventive therapy within the 6 months prior to enrollment. Patients recruited from outpatient settings were included if they had at least one symptom suggestive of TB (current cough, night sweats, fever, weight loss); inpatients were enrolled irrespective of TB symptoms.

To assess the diagnostic accuracy of FujiLAM, multiple reference standards were used as per previously published guidance [15]: a microbiological reference standard (MRS), extended MRS (eMRS), and composite reference standard (CRS) as per definitions in Table 1.

**Table 1:** List of tests and reference standard definitions^τ^.

		MRS	eMRS	CRS*
1–2 Sputum MGIT culture^Ω^		YES	YES	YES
1–2 Sputum LJ culture^Ω^		YES	YES	YES
Blood culture^Ω^		YES	YES	YES
Urine Xpert Ultra		YES	YES	YES
Sputum Xpert Ultra		YES	YES	YES
Additional (non-study) testing^§^		NO	YES	YES
2–3-month follow-up testing		YES	YES	YES
Anti-TB therapy with response		NO	NO	YES

CRS, composite reference standard; eMRS, extended microbiological reference standard; LJ, Löwenstein-Jensen; MGIT, Mycobacteria Growth Indicator Tube; MRS, microbiological reference standard. MTB, *Mycobacterium tuberculosis;* NTM, nontuberculous mycobacteria.

^Ω^ Including MTB complex confirmation and NTM determination

^§^ Any additional mycobacterial culture and/or Xpert/Ultra from other samples (e.g., pleural fluid, tissue biopsy, etc.) performed based on routine clinical indication.

*Chest X-Ray, AlereLAM and smear results might be considered as part of the clinical decision-making (as per country routine).

^τ^The respective reference standard is considered positive if any of those marked with “YES” are positive/apply. The MRS/eMRS is negative if none of the tests marked with “YES” are positive and at least one negative sputum culture is available. CRS is negative if none of those marked with “YES” are positive/apply and participant has no symptoms at 2–3-month follow-up. Unclassifiable is neither reference standard positive nor reference standard negative.

TB diagnosis in people living with HIV is challenging as standard diagnostics, such as sputum-based molecular assays, perform poorly. With low sensitivity of existing TB diagnostic tests, particularly in hospitalized PLHIV, TB is often incorrectly diagnosed and treated clinically. Therefore, complementing the microbiological reference standard with the clinical reference standard allows a stronger assessment of diagnostic performance. The reference standards used in studies often include more tests than those used in the real-world setting. The algorithm for screening and diagnosis of TB in PLHIV is dependent on whether they are screened in an inpatient or outpatient setting and described in the WHO operational handbook on tuberculosis [16, 17].

The primary objectives of the study were to determine the diagnostic accuracy of FujiLAM for TB detection among PLHIV against the eMRS and CRS. Secondary objectives included assessment of the diagnostic accuracy of FujiLAM for TB detection among PLHIV against the MRS; assessment of FujiLAM diagnostic accuracy across predefined subgroups using MRS, eMRS and CRS separately; and assessment of the diagnostic accuracy of AlereLAM individually and in comparison with FujiLAM.

Participants were invited to provide samples on the day of enrollment (Day 1) and again within 7 days of enrollment (Day 2). All participants without positive eMRS results at baseline (of samples of Day 1 and Day 2) were followed-up 2–3 months after enrollment, and an additional sample was collected if signs/symptoms (e.g. cough, fever) had not improved or completely resolved compared with baseline, as assessed by the local provider. Patients with baseline FujiLAM-positive (Day 1 and/or Day 2 urine) but negative CRS results were invited to come back at 6 months, when additional samples were collected if signs/symptoms had not improved or completely resolved.

### Procedures

**Fig 1:**
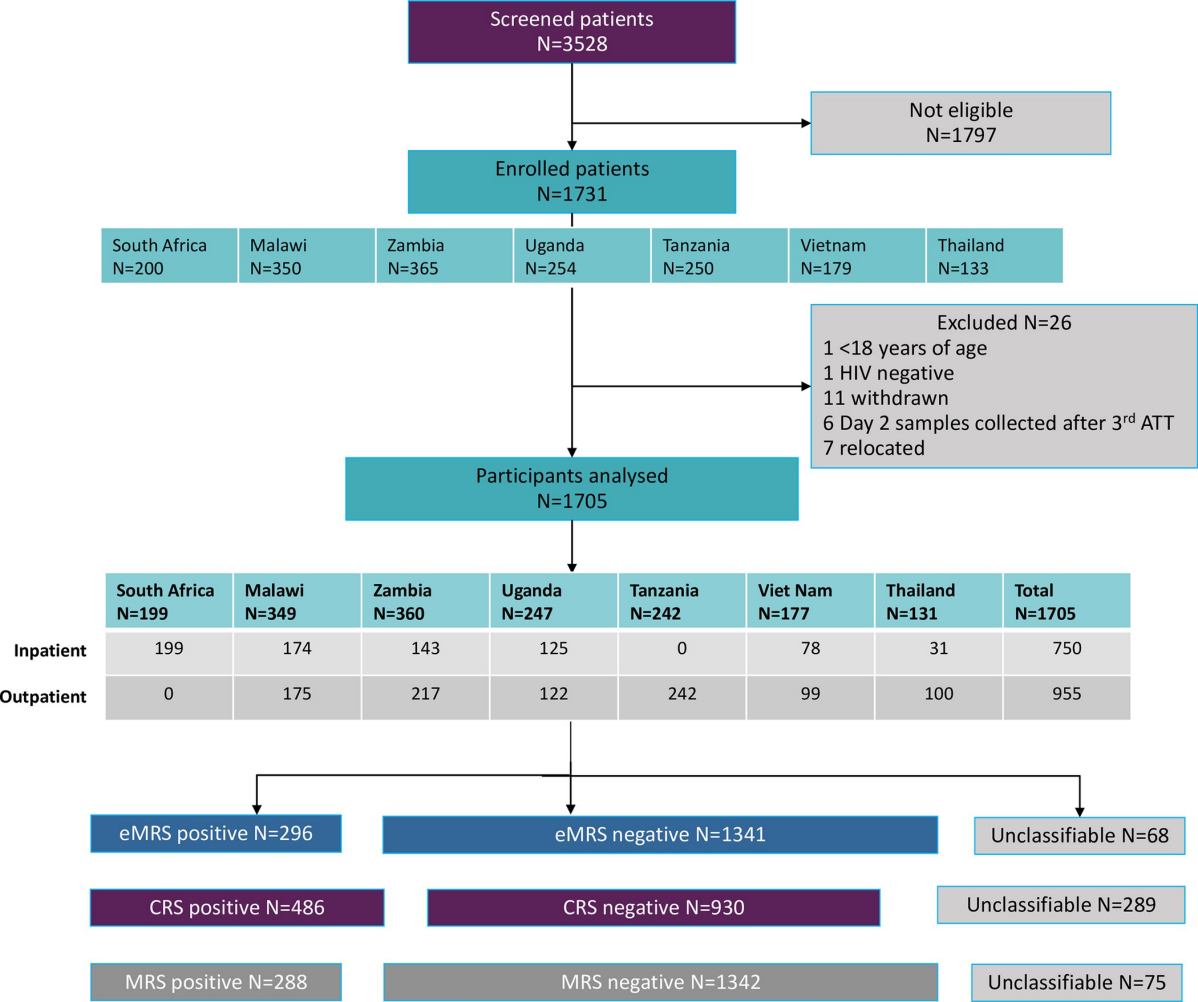
Study flow diagram. ATT, anti-TB therapy; CRS, composite reference standard; eMRS, extended microbiological reference standard; MRS, microbiological reference standard. Unclassifiable is neither reference standard positive nor reference standard negative. Reasons for non-eligibility of the 1797 persons screened but not enrolled were: being HIV negative, not interested to participate in the study, already on anti-TB treatment, on isoniazid preventive therapy, not willing to come back for follow-up visit, too weak or confused, refused to give blood.

The testing flow and number of samples tested are shown in Fig 1 in S5 File.

Urine, sputum and blood specimens were collected and processed fresh from participants after informed consent was obtained and clinical assessment was completed. Urine specimens were collected on the day of enrollment (spot urine), within 7 days of enrollment (early morning) and at the 6-month follow-up visit (if indicated, further details in S2 File). Urine samples were tested using FujiLAM and AlereLAM, and remaining urine samples were submitted for Xpert MTB/RIF Ultra (Cepheid, Sunnyvale, CA, USA) testing. For this, 30 ml urine was centrifuged at 3000x g for 15 minutes and, following removal of the supernatant, the pellet was re-suspended in 0.75 ml phosphate-buffered saline and 1·5 ml sample reagent buffer. Subsequently, 2 ml of the reagent-treated specimen was tested. When possible, left-over urine was preserved at ˗80 ⁰C on-site for additional testing.

Blood was collected on Day 1 and submitted for CD4 cell count (flow cytometry) and mycobacterial blood culture. Sputum samples were collected on study Days 1 (spot) and 2 (early morning), and at the 3- and 6-month visits (if indicated) and tested by smear microscopy (fluorescence microscopy using Auramine O staining and/or Ziehl-Neelsen staining), Mycobacteria Growth Indicator Tube liquid culture (MGIT; Becton Dickinson, Franklin Lakes, NJ, USA), solid culture on Löwenstein-Jensen (LJ) medium, and Xpert MTB/RIF Ultra. If a participant was unable to provide sputum spontaneously, an attempt was made to collect induced sputum (depending on site regulations, participant health status, and COVID-19 restrictions).

Speciation was done from any positive mycobacterial culture (sputum, blood) using MPT64 antigen detection and/or MTBDR*plus*, MTBC and CM/AS line probe assays (Hain Lifescience, Nehren, Germany). Blood culture from all participants were done in BACTEC™ Myco/F Lytic culture vials (Becton Dickinson, Franklin Lakes, NJ, USA). WHO prequalified in vitro rapid diagnostic tests were used for HIV testing. Chest X-ray was performed on the day of enrollment if not done already by the treating clinical team. Day 1 samples were collected on the day of enrollment and tested within 24 hours; Day 2 samples were collected within seven days of enrollment and tested within 24 hours. Additional non-study samples were collected at the discretion of the treating clinician.

### FujiLAM testing

Testing with the investigational product, FujiLAM, was performed at POC (either bedside/adjacent room a few meters from the ward/clinic) according to manufacturer’s instructions on the day of collection [11, 18]; ideally within 2 hours of sample collection, or samples were kept at 2–8°C until testing occurred.

The FujiLAM tests used in this study were CE marked and manufactured under ISO13485 by Fujifilm. Quality control requirements for lot release were determined and assessed by the manufacturer (Fujifilm). Quality control was not done for the incoming lots, as no reference/control material was provided by the manufacturer.

### AlereLAM testing

AlereLAM testing was performed at POC as per manufacturer’s instructions, using the test’s Reference Scale Card of 4 grades with the Grade 1 cut-off point as the positivity threshold.

AlereLAM and FujiLAM tests were done for each patient at Day 1, Day 2, and 6-months follow-up (if indicated). Both tests were performed and interpreted independently by different operators to ensure blinding. Operators were trained prior to the study start and their competency was assessed. Initially, operators were trained by master trainers on site (in Malawi, South Africa, Uganda, and Tanzania). This training included presentation slides, on-site practicing and user competency assessment (as per the proficiency testing tools for FujiLAM and AlereLAM provided in S3 and S4 Files, respectively). Following COVID-19-related travel restrictions, training of trainer-operators took place online via presentations, with demonstration/observation via the camera and competency assessment (in Zambia, Viet Nam, and Thailand). Those trainer-operators then trained other operators, completed their competency assessment and shared videos for master trainers to review. Operator profiles varied between and within countries, but could include field workers, nurses, and medical officers.

Operators were also blinded to reference tests and all results obtained from other tests. Laboratory personnel performing the reference standard testing did not have access to the results of the FujiLAM and AlereLAM tests. Results of FujiLAM tests were not communicated to the managing clinical team, but AlereLAM results were, if AlereLAM formed part of the local country guidelines of the study site (Malawi, South Africa, Tanzania, Thailand, Uganda, and Zambia).

Invalid FujiLAM or AlereLAM tests were repeated once. Further details of testing and operator training for both tests are included in S3 and S4 Files. FujiLAM and AlereLAM proficiency tools.

### Reference standard testing

For reference standard testing, the specimens were processed using standardized protocols from centralized accredited laboratories of the different partner sites. Sputum, blood, and urine specimens were collected to ensure comprehensive reference standards.

Reference standard positives and negatives were defined as per Table 1 in S5 File. Results from additional non-study specimens were captured for eMRS classification. Baseline reference standards considered test results available from Day 1 and/or Day 2 samples.

### Post-hoc assessment of lot-to-lot variability

Variability across the six FujiLAM lots was assessed at the Research Institute of Tuberculosis of the Japan Anti-Tuberculosis Association (RIT-JATA, Tokyo, Japan). For this post-hoc analysis 181 urine samples were selected: 111 were FujiLAM positive but eMRS negative from this study (Table 2 in S5 File) and an additional 70 were well-characterized samples from the FIND biobank of 50 microbiologically confirmed TB and 20 non-TB patients (see the procedures section in the online data supplement for more detail). All 181 urine samples were tested on each of the six FujiLAM lots and the AlereLAM test in singlets. Each test was interpreted by two operators independently and in case of discordant results, the operators re-inspected the test strip together to establish the final consensus result through mutual agreement. Operators were blinded to the initial result of the 181 samples. LAM concentration was further quantified using the ultrasensitive laboratory-based electrochemiluminescence LAM assay (EclLAM, Meso Scale Diagnostics, Rockville, MD, USA) employing the same antibody pair as the FujiLAM assay [12, 19].

### Statistics

A sample size of 233 confirmed TB patients across all sites was considered adequate to obtain an estimate of 60% (+/˗9%) overall FujiLAM sensitivity [11] with 95% Wilson’s Confidence Interval (CI), 80% power and 5% alpha.

Descriptive statistics were used to characterize participants. Index test sensitivity and specificity were determined using MRS, eMRS and CRS as reference standards. Overall sensitivity and specificity were calculated by pooling results from different sites. Results are presented with 95% CI based on Wilson’s score method [20].

Patients with invalid LAM-based test results and/or with all reference test results being contaminated or invalid were excluded from the relevant analyses.

Data analysis was performed with R (version 4·1·2) based on a predefined statistical analysis plan and reported according to STARD guidelines [21]. The statistical analysis plan is available upon request. The performance analysis by lot was done post-hoc.

Generalized linear mixed models (GLMM) were constructed post-hoc to investigate factors contributing to the variation in the agreement (match/mismatch) between the reference (eMRS) and FujiLAM using “lme4” package, “glmer” function, with a binomial error distribution [22]. Age, sex, country, lot, visit (Day 1/Day 2), CD4 counts (log-transformed), urine colour, urine turbidity, and hospitalization setting (inpatient/outpatient) were included as fixed effects, patient ID was included as a random effect, and the test reader was included as a random effect nested within the country. Model summaries include fixed effect coefficients, standard errors, z-values and associated p-values, odds ratios, and their 95% CIs. Adjusted P values were calculated using the Benjmini-Hochberg method [23].

### Ethics statement

All study-related activities were approved by each country’s Research Ethics Committee (details in Table 1 in S5 File). Written informed consent was obtained from participants, as per the study protocol. Study participation did not affect standard of care. The full study protocol is available at clinicaltrials.gov (NCT04089423). Additional information regarding the ethical, cultural, and scientific considerations specific to inclusivity in global research is included in the S7 File.

## Results

Across the study sites, 3528 PLHIV at risk of having pulmonary and/or extra-pulmonary TB were screened for eligibility. Of these, 1731 participants consented to participate in the study (Fig 1).

A total of 1637 participants had results for all index and eMRS tests available and were included in the analysis. Table 2 shows their baseline demographic and clinical characteristics. The median age was 40 years (range: 18–82 years) and 52% were female. Overall, 26% had a history of prior TB treatment, 5% had a history of prior ART and 78% were on ART at the date of consent. Median CD4 count was 369 cells/μl. Of the 1637 participants, 296 (18%) were classified as positive for TB by eMRS and 1341 (81.9%) as negative for TB. Table 3 in S5 File shows the demographic and clinical characteristics of the study participants stratified by country.

**Table 2:** Demographic and clinical characteristics of study participants.

* *	All	TB	Non-TB
N	1637	296	1341
Age median [min-max] (years)	40 [18–82]	39.5 [19–75]	41 [18–82]
Age [IQR]	40 [15]	39.5 [13.25]	41 [15]
Female, no. (%)	852/1637 (52)	139/296 (47)	713/1341 (53)
Median CD4 count—cells/μl [min-max]	369 [0–3643]	205 [1–1464]	402.5 [0–3643]
CD4 count—cells/μl [IQR]	369 [493]	205 [431.5]	402.5 [484.75]
Seriously ill, no. (%)	217/1637 (13)	82/296 (28)	135/1341 (10)
History of TB, no. (%)	426/1637 (26)	88/296 (30)	338/1341 (25)
WHO TB Symptoms, no. (%)	1521/1637 (93)	290/296 (98)	1231/1341 (92)
TB prevalence, no. (%)	296/1637 (18)	296/296 (100)	0/1341 (0)
***Setting***	*** ***	*** ***	*** ***
Inpatients, no. (%)	684/1637 (42)	146/296 (49)	538/1341 (40)
Outpatients, no. (%)	953/1637 (58)	150/296 (51)	803/1341 (60)
***CD4***	*** ***	*** ***	*** ***
≤100	335/1637 (20)	102/296 (34)	233/1341 (17)
>100 to ≤200	186/1637 (11)	41/296 (14)	145/1341 (11)
>200 to ≤500	508/1637 (31)	78/296 (26)	430/1341 (32)
>500	592/1637 (36)	70/296 (24)	522/1341 (39)
CD4 Unknown	16/1637 (1)	5/296 (2)	11/1341 (1)
Seriously ill—CD4 ≤100, no. (%)	102/335 (30)	47/102 (46)	55/233 (24)
Seriously ill—CD4 ≤200, no. (%)	131/521 (25)	57/143 (40)	74/378 (20)
***HIV treatment***	*** ***	*** ***	*** ***
ART in the past, no. (%)	90/1637 (5)	26/296 (9)	64/1341 (5)
Currently on ART, no. (%)	1272/1637 (78)	203/296 (69)	1069/1341 (80)
Don t know, no. (%)	18/1637 (1)	3/296 (1)	15/1341 (1)
Never used, no. (%)	257/1637 (16)	64/296 (22)	193/1341 (14)
***Speciation***	*** ***	*** ***	*** ***
NTM, no. (%)	102/253 (40)	15/166 (9)	87/87 (100)
NTM/MTBC, no. (%)	6/253 (2)	6/166 (4)	0/87 (0)
MTBC, no. (%)	145/253 (57)	145/166 (87)	0/87 (0)
***Follow-up status***	*** ***	*** ***	*** ***
Died within 3 months, no. (%)	126/1637 (8)	31/296 (10)	95/1341 (7)
Alive, no. (%)	1250/1637 (76)	123/296 (42)	1127/1341 (84)
Lost to follow-up, no. (%)	124/1637 (8)	10/296 (3)	114/1341 (8)
No follow-up done, no. (%)	137/1637 (8)	132/296 (45)	5/1341 (0)

*Seriously ill if any of the followings present: respiratory rate > 30 breaths/min, heart rate > 120 beats/min, body mass index [BMI] ≤ 18.5 kg/m2, systolic blood pressure < 90 mmHg or being unable to walk unaided

ART, antiretroviral therapy; FU, follow-up; MTBC, Mycobacterium tuberculosis complex; no. number; NTM, non-tuberculous mycobacteria; TB, tuberculosis; WHO, World Health Organization.

### Diagnostic accuracy of FujiLAM

Overall sensitivity of FujiLAM against the eMRS on Day 1 was 54·4% (95% CI: 48·7–60·0), with an overall specificity of 85·2% (83·2–87·0) (Table 3). The Day 2 early morning sample had lower sensitivity and specificity estimates: 51·0% (45·3–56·7) and 81·8% (79·6–83·8), respectively (Table 4 in S5 File). In comparison, overall sensitivity of AlereLAM against the eMRS on Day 1 was 30·3% (25·3–35·8), with an overall specificity of 90·7% (89·0–92·2). On Day 2 early morning urine samples, the sensitivity of AlereLAM was 28.2% (23·3–33·6) and specificity was 87·5% (85·7–89·2) (Table 5 in S5 File).

**Table 3:** Sensitivity and specificity of Day 1 FujiLAM against the eMRS.

	N	TP	FP	FN	TN	Sensitivity [95%CI]	Specificity [95%CI]
All	1628	160	197	134	1137	54.4 [48.7–60.0]	85.2 [83.2–87.0]
***CD4***	*** ***						
≤100	331	83	44	18	186	82.2 [73.6–88.4]	80.9 [75.3–85.4]
101 to ≤200	184	25	18	15	126	62.5 [47.0–75.8]	87.5 [81.1–91.9]
201 to ≤500	507	35	70	43	359	44.9 [34.3–55.9]	83.7 [79.9–86.9]
>500	590	15	64	55	456	21.4 [13.4–32.4]	87.7 [84.6–90.2]
Unknown	16	2	1	3	10	40.0 [11.8–76.9]	90.9 [62.3–98.4]
***Setting***	*** ***						
Inpatient	677	100	63	44	470	69.4 [61.5–76.4]	88.2 [85.2–90.6]
Outpatient	951	60	134	90	667	40.0 [32.5–48.0]	83.3 [80.5–85.7]
***Country***	*** ***						
South Africa	144	41	22	15	66	73.2 [60.4–83.0]	75.0 [65.0–82.9]
Malawi	334	20	36	12	266	62.5 [45.2–77.1]	88.1 [83.9–91.3]
Zambia	358	33	56	20	249	62.3 [48.8–74.1]	81.6 [76.9–85.6]
Uganda	246	32	36	12	166	72.7 [58.1–83.7]	82.2 [76.3–86.8]
Tanzania	242	18	23	50	151	26.5 [17.4–38.0]	86.8 [81.0–91.0]
Vietnam	176	11	5	22	138	33.3 [19.8–50.4]	96.5 [92.1–98.5]
Thailand	128	5	19	3	101	62.5 [30.6–86.3]	84.2 [76.6–89.6]

The overall sensitivity of FujiLAM against the CRS on Day 1 was 45·0% (95% CI: 40·7–49·5), with an overall specificity of 86·5% (84·2–88·6) (Tables 6 in S5 File).

Subsequent sub-group analyses for Day 1 samples against eMRS, Day 2 results and estimates against MRS and CRS are reported in the Tables 4, 6, and 8 in S5 File.

Stratified by CD4 count, FujiLAM sensitivity was 82·2% (73·6–88·4) and specificity was 80·9% (75·3–85·4) in participants with a CD4 count ≤100 cells/μl; sensitivity decreased at higher CD4 count strata, while specificity varied as shown in Table 3. In participants with CD4 201–500 cells/μl, sensitivity of FujiLAM was 44·9% (34·3–55·9) and specificity was 83·7% (79·9–86·9), while with CD4>500 cells/μl sensitivity was 21·4% (13·4–32·4) with 87·7% (84·6–90·2) specificity (Table 3).

When stratified by setting, for inpatients, sensitivity of FujiLAM was 69·4% (61·5–76·4) and specificity was 88·2% (85·2–90·6). However, for outpatients, the tests showed reduced sensitivity and specificity at 40·0% (32·5–48·0) and 83·3% (80·5–85·7), respectively (Table 3).

**Fig 2:**
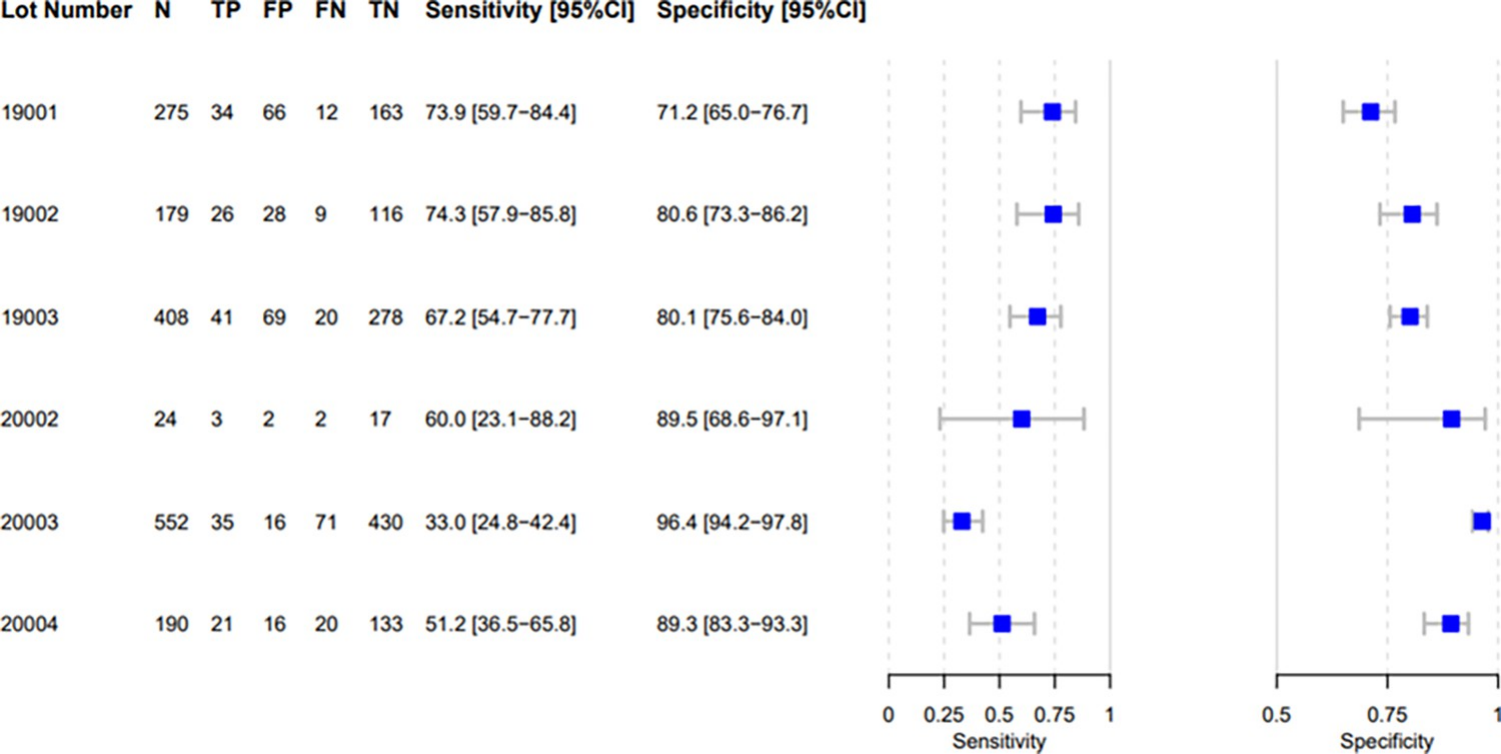
FujiLAM performance by lot. Out of the total of 1637 participants, nine did not have a valid FujiLAM result on Day 1 and were not included in the FujiLAM Day 1 diagnostic accuracy analysis. FN, false negative; FP, false positive; N, number; TN, true negative; TP true positive.

Accuracy estimates varied considerably between countries, with sensitivity ranging from 26·5% (Tanzania), to 73.2 (South Africa), and specificity from 75·0% (South Africa) to 96·5% (Viet Nam, Table 3). The same variability was observed across the different reference standards (MRS, CRS). Because the lot distribution was uneven between countries and could explain these differences (Fig 2 in S5 File), in post-hoc analysis we calculated FujiLAM accuracy by lot. This identified substantial FujiLAM lot-to-lot variability, with certain lots delivering low specificity/high sensitivity and others delivering high specificity/low sensitivity (Fig 2).

The diagnostic accuracy analysis of AlereLAM results on Day 1 and Day 2 against eMRS, CRS and MRS are reported in Tables 5, 7 and 9 in S5 File, respectively. These analyses also reveal country-specific differences, however, to a lesser degree than for FujiLAM. Table 10 in S5 File shows the number of additional microbiological tests per country considered for the eMRS, done by the routine clinical team.

### Additional post-hoc exploratory analysis

The regression model suggested that the factors significantly contributing to the variation between reference standard and FujiLAM were lot number (χ^2^_5_ = 51·4 *p* = 7·16x10^-10^), countries (χ^2^_6_ = 38·8, *p* = 7·81x10^-7^) and visit days (χ^2^_1_ = 9·10, *p* = 0·0025) (Tables 11 and 12 in S5 File). However, because lots were not evenly distributed across countries, these factors may be interdependent, and the variation between different countries may be explained by the variation between lots or vice versa.

**Fig 3:**
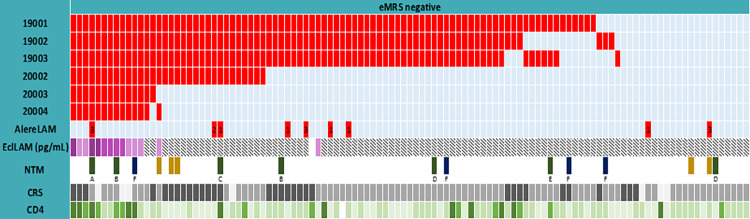
Exploratory comparison of FujiLAM positivity rates in 111 eMRS negative, FujiLAM positive Day 1 samples from the study. eMRS, extended microbiological reference standard; NTM, nontuberculous mycobacteria; CRS, composite reference standard; Red cells indicate positive result and light blue cells indicate negative result on FujiLAM or AlereLAM. For AlereLAM positive results, the numbers further indicate line grade intensity (1–3). LAM concentration measured with EclLAM is illustrated on the purple scale from darkest to lightest: >200 pg/mL, 51–200 pg/mL, 11–50 pg/mL; diagonal stipe pattern indicates <limit of detection (11 pg/mL); Dark yellow–Full NTM speciation not done; green—slow growing mycobacteria; dark blue- fast growing mycobacteria; A- *M*. *simiae*; B- *M*. *intracellulare*; C- *M*. *avium*, D- *M*. *scrofulaceum*; D- *M*. *gordonae*; F- *M*. *fortuitum;* Dark grey- CRS positive, mid grey-CRF neg, light grey- unclassifiable. Very dark green: CD4 counts of ≤100 cells/μl; dark green: CD4 counts of 101–200 cells/μl; mid green: CD4 counts of 201–500 cells/μl; light green: CD4 counts of ≥500 cells/μl; white: no CD4 data available.

For eMRS positive patients (focusing on sensitivity), the only factor that remained significant was CD4 count (Tables 13 and 14 in S5 File), where higher CD4 count was associated with a higher mismatch ratio (see Fig 3 in S5 File). However, for eMRS negative patients (focusing on specificity), lot remained the most significant factor (χ^2^_5_ = 97·2, *p*<2·02x10^-19^), thus explaining the variation in agreement between the reference standard and the FujiLAM test (Tables 15 and 16 in S5 File).

To verify the impact of lots on performance, we analysed 111 FujiLAM-positive (Day 1 samples), eMRS-negative urine specimens from the study on all six lots used in the study and with AlereLAM. As shown in Fig 3 and Table 17 in S5 File, FujiLAM positivity rates varied from 14/111 (13%) to 86/111 (77%) between lots. In addition, we quantified the concentration of LAM in 110 of the 111 samples using EclLAM (one sample was not available for EclLAM testing due to insufficient volume). A total of 14 samples had measurable LAM concentration (>11 pg/mL); of these, 12 were concordant positive on all six FujiLAM lots tested, of which three were further classified as CRS positive, eight as CRS negative and three as CRS unclassifiable (patient passed away). Five of these 12 had CD4 ≤100 cells/μl, four had CD4 101–200 cells/μl while three had CD4 201–500 cells/μl. Twenty-one of the 111 samples tested negative on all six FujiLAM lots.

Non-tuberculous mycobacteria (NTM) have been found to cross-react with LAM, which may cause false-positive results in patients with NTM infection without TB [24, 25]. However, based on the data available from this study, there is no clear indication that false positivity correlates with the presence of NTM infection measured in sputum samples. Four of the eight CRS-negative, FujiLAM-positives had LAM levels above the detection limit of the EclLAM assay and had NTM detected, but notably from a non-sterile sputum sample only; two were slow-growing, one was fast-growing, and one was non-specified NTM.

We furthermore determined FujiLAM (six lots) and AlereLAM positivity rates in a series of well-characterized biobank specimens from 50 patients with microbiologically confirmed TB and 20 patients with negative microbiological test results (Table 18 in S5 File). This experiment confirmed that high positivity rates were associated with certain lots.

## Discussion

This prospective, multi-centre diagnostic accuracy study of the FujiLAM test in PLHIV was conducted among inpatient and outpatient settings in seven countries across sub-Saharan Africa and Asia. We observed considerably higher sensitivity of the FujiLAM test than AlereLAM; however, its specificity was lower than expected from previously published studies [11, 14, 26]. Furthermore, we observed a large variability in FujiLAM sensitivity and specificity between countries against all the reference standards, which was attributed to variable performance between FujiLAM lots, limiting our interpretation and generalizability of study findings.

Our main finding of lot-to-lot variability was also observed in a separate multicentre study conducted on HIV positive patients aged ≥15 years using four of the six lots used in this study. That analysis was triggered by our findings when presented in a preprint of this paper [27, 28]. What this present paper adds to the body of evidence is the systematic capturing of lot information and the post-hoc exploratory study confirming the suspected lot-to-lot variability issue. A recent meta-analysis, including five adult cohorts of PLHIV from three countries using three different lots (98002, 98004, 98006), found no inconsistency in diagnostic accuracy [14]. In another study from Nigeria, sub-group analysis of FujiLAM performance by HIV status, using lot 20001, showed 93·3% specificity [29], comparable to previous studies and to the specificity values of lots 20002, 20003 and 20004 used in this study.

Assessment of lot variability was not considered in the conceptualization of the study; however, the large study size allowed for exploratory analysis of factors that could explain the unexpected clinical variability. Given the patient heterogeneity in CD4 count, disease severity and other country effects, it is difficult to assess the lot effect in isolation in the clinical trial. We therefore did a post-hoc re-analysis of 111 of the 197 samples deemed false positive in the clinical study from sites with local biobanks and ethical approval for reanalysis abroad, and an additional 70 representative samples from the FIND biobank. This sub-study confirmed a significant difference in positivity between the six lots used in the study in both the banked samples and the rerun samples from the trial. EclLAM, a quantitative research assay employing the same antibodies as FujiLAM, only detected LAM in 14 of the 110 eMRS-negative FujiLAM-positive samples from the study. Twenty-one of the 111 samples tested negative on all six FujiLAM lots, all of these had LAM concentration measured by EclLAM below the limit of detection.

Overall, we found higher specificity of AlereLAM compared with FujiLAM (a difference of 5.5%), and we observed some variability of AlereLAM specificity at country level (85%–99%), although much less compared with FujiLAM (75–97% depending on lot used). Previously published studies in PLHIV found consistently lower specificity of FujiLAM compared with AlereLAM for those with CD4 <200 cells/μL (a difference of 13.1% for CD4 0–100; and 1.7% for CD4 100–200) [11, 14, 26]. This was not observed in PLHIV with high CD4 cell counts or in patients without HIV [12] and has been interpreted in parts to an effect driven by the imperfect reference standard for TB diagnosis, which disproportionally affects more sensitive tests and results in lower specificity [11, 14, 30, 31].

As the FujiLAM test is visually read and interpreted, it is not possible to adjust interpretation of specific lots, as with most lateral flow tests using a reference card (such as AlereLAM) [32] or computerized reader. Current and future LAM tests may benefit from a reading device, which could improve consistency and remove reader subjectivity, particularly for bands close to the low cut-off in the pg/ml range, which is required for LAM tests to reach sufficient sensitivity [19]. A reader device with connectivity can further enable automated linkage of test results to care, as well as improved surveillance.

The observed FujiLAM lot variability can impact patient management. Translating the findings to a setting with 10% prevalence of TB among patients presenting for care, the most extreme performing lots, 19001 (sensitivity: 73·9 [59·7–84·4]; specificity: 71·2 [65·0–76·7]) and 20003 (sensitivity: 33·0 [24·8–42·4]; specificity: 96·4 [94·2–97·8]) would render large differences in test outcomes. For each 1000 tested individuals, lot 19001 would identify 74 (95% CI: 60–84) true positive and 26 (95% CI: 16 to 40) false negatives, whereas lot 20003 would identify 33 (25–42) true positive and 67 (58–75%) false negatives. More worryingly, lot 19001 would identify 259 (210–315) false positives, whereas lot 20003 would only identify 32 (21–52) false positives (Tables 20 and 21 in S5 File). This variability in both sensitivity and specificity is unacceptably high for clinical management of patients.

Several ongoing studies are evaluating the accuracy of FujiLAM and specific lot analyses will be important to verify the findings from this study. Altogether, this study underlines the importance of conducting manufacturer-independent evaluations of new diagnostic tests. When designing a diagnostic accuracy study, it is critical to include at least two lots evenly distributed across the clinical sites and systematic quality control using external reference material. To our knowledge, there are currently no available quality assessment panels for a LAM-based test.

In conclusion, this large multi-country clinical trial of the diagnostic accuracy of the FujiLAM test observed higher FujiLAM sensitivity in PLHIV with low CD4 cell counts and in inpatients, in accordance with previous studies. However, specificity was lower than expected, and accuracy estimates were variable and associated with specific FujiLAM lots, as confirmed through additional post-hoc testing and analysis. The lot variability issue with the FujiLAM test is a major setback in the quest towards a POC non-sputum-based TB test. Although the results obtained using the current version of the FujiLAM test are too variable for clinical decision-making, a new version of the test (work already undergoing by the manufacturer) could improve POC testing for TB diagnosis in PLHIV. Despite these challenges and unexpected observations, it is important to emphasize the promise that the LAM biomarker and LAM tests hold for TB testing.

## Supporting information

S1 File TREND checklist. (DOCX)

S2 File Supplementary methods and results. (DOCX)

S3 File FujiLAM proficiency tool. (DOCX)

S4 File AlereLAM proficiency tool. (DOCX)

S5 File Figures and tables. (DOCX)

S6 File Supplementary references. (DOCX)

S7 File Inclusivity in global research. (DOCX)

S8 File FujiLAM study consortium members. (DOCX)

## References

[1] World Health Organization. Global Tuberculosis Report 2021. 2021.

[2] World Health Organization. Tuberculosis 2022 [1 June 2023]. Available from: https://www.who.int/news-room/fact-sheets/detail/tuberculosis#:~:text=Key%20facts,with%20tuberculosis%20(TB)%20worldwide.

[3] Getahun H, Ford N. Tackling the persistent burden of tuberculosis among people living with HIV. J Int AIDS Soc. 2016;19(1):21002. Epub 2016/03/29. doi: 10.7448/IAS.19.1.21002 ; PubMed Central PMCID: PMC4808692. 27018421 PMC4808692

[4] Gupta-Wright A, Corbett EL, van Oosterhout JJ, Wilson D, Grint D, Alufandika-Moyo M, et al. Rapid urine-based screening for tuberculosis in HIV-positive patients admitted to hospital in Africa (STAMP): a pragmatic, multicentre, parallel-group, double-blind, randomised controlled trial. Lancet. 2018;392(10144):292–301. Epub 2018/07/24. doi: 10.1016/S0140-6736(18)31267-4 ; PubMed Central PMCID: PMC6078909. 30032978 PMC6078909

[5] Peter JG, Zijenah LS, Chanda D, Clowes P, Lesosky M, Gina P, et al. Effect on mortality of point-of-care, urine-based lipoarabinomannan testing to guide tuberculosis treatment initiation in HIV-positive hospital inpatients: a pragmatic, parallel-group, multicountry, open-label, randomised controlled trial. The Lancet. 2016;387(10024):1187–97. doi: 10.1016/S0140-6736(15)01092-2 26970721

[6] Gupta RK, Lucas SB, Fielding KL, Lawn SD. Prevalence of tuberculosis in post-mortem studies of HIV-infected adults and children in resource-limited settings: a systematic review and meta-analysis. Aids. 2015;29(15):1987–2002. Epub 2015/08/13. doi: 10.1097/QAD.0000000000000802 ; PubMed Central PMCID: PMC4568896. 26266773 PMC4568896

[7] Huerga H, Ferlazzo G, Bevilacqua P, Kirubi B, Ardizzoni E, Wanjala S, et al. Incremental Yield of Including Determine-TB LAM Assay in Diagnostic Algorithms for Hospitalized and Ambulatory HIV-Positive Patients in Kenya. PLoS One. 2017;12(1):e0170976. Epub 2017/01/27. doi: 10.1371/journal.pone.0170976 ; PubMed Central PMCID: PMC5268475. 28125693 PMC5268475

[8] Boyles TH, Griesel R, Stewart A, Mendelson M, Maartens G. Incremental yield and cost of urine Determine TB-LAM and sputum induction in seriously ill adults with HIV. Int J Infect Dis. 2018;75:67–73. Epub 2018/08/21. doi: 10.1016/j.ijid.2018.08.005 ; PubMed Central PMCID: PMC6202059. 30125689 PMC6202059

[9] García JI, Mambuque E, Nguenha D, Vilanculo F, Sacoor C, Sequera VG, et al. Mortality and risk of tuberculosis among people living with HIV in whom TB was initially ruled out. Sci Rep. 2020;10(1):15442. Epub 2020/09/24. doi: 10.1038/s41598-020-71784-3 ; PubMed Central PMCID: PMC7509810. 32963296 PMC7509810

[10] World Health Organization. High-priority target product profiles for new tuberculosis diagnostics: report of a consensus meeting 2014 [28 January 2022]. Available from: https://apps.who.int/iris/handle/10665/135617.

[11] Broger T, Sossen B, du Toit E, Kerkhoff AD, Schutz C, Ivanova Reipold E, et al. Novel lipoarabinomannan point-of-care tuberculosis test for people with HIV: a diagnostic accuracy study. Lancet Infect Dis. 2019;19(8):852–61. Epub 2019/06/04. doi: 10.1016/S1473-3099(19)30001-5 ; PubMed Central PMCID: PMC6656794. 31155318 PMC6656794

[12] Broger T, Nicol MP, Sigal GB, Gotuzzo E, Zimmer AJ, Surtie S, et al. Diagnostic accuracy of 3 urine lipoarabinomannan tuberculosis assays in HIV-negative outpatients. J Clin Invest. 2020;130(11):5756–64. Epub 2020/07/22. doi: 10.1172/JCI140461 ; PubMed Central PMCID: PMC7598043. 32692731 PMC7598043

[13] Bulterys MA, Wagner B, Redard-Jacot M, Suresh A, Pollock NR, Moreau E, et al. Point-Of-Care Urine LAM Tests for Tuberculosis Diagnosis: A Status Update. J Clin Med. 2019;9(1). Epub 2020/01/08. doi: 10.3390/jcm9010111 ; PubMed Central PMCID: PMC7020089. 31906163 PMC7020089

[14] Broger T, Nicol MP, Székely R, Bjerrum S, Sossen B, Schutz C, et al. Diagnostic accuracy of a novel tuberculosis point-of-care urine lipoarabinomannan assay for people living with HIV: A meta-analysis of individual in- and outpatient data. PLoS Med. 2020;17(5):e1003113. Epub 2020/05/02. doi: 10.1371/journal.pmed.1003113 . 32357197 PMC7194366

[15] Drain PK, Gardiner J, Hannah H, Broger T, Dheda K, Fielding K, et al. Guidance for Studies Evaluating the Accuracy of Biomarker-Based Nonsputum Tests to Diagnose Tuberculosis. The Journal of Infectious Diseases. 2019;220(Supplement_3):S108–S15. doi: 10.1093/infdis/jiz356 31593598

[16] World Health Organization. WHO consolidated guidelines on tuberculosis. Module 3: Diagnosis—Rapid diagnostics for tuberculosis detection 2021 update 2021 [3 February 2022]. Available from: https://www.who.int/publications/i/item/9789240029415. 34314130

[17] World Health Organization. WHO operational handbook on tuberculosis: module 2: screening: systematic screening for tuberculosis disease. 2022. 33822560

[18] FIND. Fujifilm SILVAMP TB LAM test procedure. YouTube 2019.

[19] Sigal GB, Pinter A, Lowary TL, Kawasaki M, Li A, Mathew A, et al. A Novel Sensitive Immunoassay Targeting the 5-Methylthio-d-Xylofuranose-Lipoarabinomannan Epitope Meets the WHO’s Performance Target for Tuberculosis Diagnosis. J Clin Microbiol. 2018;56(12). Epub 2018/09/28. doi: 10.1128/JCM.01338-18 ; PubMed Central PMCID: PMC6258851. 30257899 PMC6258851

[20] Wilson EB. Probable Inference, the Law of Succession, and Statistical Inference. Journal of the American Statistical Association. 1927;22(158):209–12. doi: 10.1080/01621459.1927.10502953

[21] Bossuyt PM, Reitsma JB, Bruns DE, Gatsonis CA, Glasziou PP, Irwig L, et al. STARD 2015: An Updated List of Essential Items for Reporting Diagnostic Accuracy Studies. Clin Chem. 2015;61(12):1446–52. Epub 2015/10/30. doi: 10.1373/clinchem.2015.246280 . 26510957

[22] Bates D, Mächler M, Bolker B, Walker S. Fitting Linear Mixed-Effects Models Using lme4. Journal of Statistical Software. 2015;67(1):1–48. doi: 10.18637/jss.v067.i01

[23] Benjamini Y, Hochberg Y. Controlling the False Discovery Rate: A Practical and Powerful Approach to Multiple Testing. Journal of the Royal Statistical Society: Series B (Methodological). 1995;57(1):289–300. 10.1111/j.2517-6161.1995.tb02031.x.

[24] Nel JS, Lippincott CK, Berhanu R, Spencer DC, Sanne IM, Ive P. Does Disseminated Nontuberculous Mycobacterial Disease Cause False-Positive Determine TB-LAM Lateral Flow Assay Results? A Retrospective Review. Clinical Infectious Diseases. 2017;65(7):1226–8. doi: 10.1093/cid/cix513 28575238

[25] Qvist T, Johansen IS, Pressler T, Høiby N, Andersen AB, Katzenstein TL, et al. Urine lipoarabinomannan point-of-care testing in patients affected by pulmonary nontuberculous mycobacteria—experiences from the Danish Cystic Fibrosis cohort study. BMC Infect Dis. 2014;14:655. Epub 2014/12/05. doi: 10.1186/s12879-014-0655-4 ; PubMed Central PMCID: PMC4260379. 25471640 PMC4260379

[26] Bjerrum S, Broger T, Székely R, Mitarai S, Opintan JA, Kenu E, et al. Diagnostic Accuracy of a Novel and Rapid Lipoarabinomannan Test for Diagnosing Tuberculosis Among People With Human Immunodeficiency Virus. Open Forum Infect Dis. 2020;7(1):ofz530. Epub 2020/01/25. doi: 10.1093/ofid/ofz530 ; PubMed Central PMCID: PMC6966242. 31976353 PMC6966242

[27] Huerga H, Bastard M, Lubega AV, Akinyi M, Antabak NT, Ohler L, et al. Novel FujiLAM assay to detect tuberculosis in HIV-positive ambulatory patients in four African countries: a diagnostic accuracy study. Lancet Glob Health. 2023;11(1):e126–e35. Epub 2022/12/16. doi: 10.1016/S2214-109X(22)00463-6 ; PubMed Central PMCID: PMC9747168. 36521944 PMC9747168

[28] Székely R, Sossen B, Mukoka M, Muyoyeta M, Nakabugo E, Hella J, et al. Multicentre accuracy trial of FUJIFILM SILVAMP TB LAM test in people with HIV reveals lot variability. medRxiv. 2022:2022.09.07.22278961. doi: 10.1101/2022.09.07.22278961 PMC11142480 38820372

[29] Comella-Del-Barrio P, Bimba JS, Adelakun R, Kontogianni K, Molina-Moya B, Osazuwa O, et al. Fujifilm SILVAMP TB-LAM for the Diagnosis of Tuberculosis in Nigerian Adults. J Clin Med. 2021;10(11). Epub 2021/07/03. doi: 10.3390/jcm10112514 ; PubMed Central PMCID: PMC8201264. 34204120 PMC8201264

[30] Huerga H, Rucker SCM, Bastard M, Dimba A, Kamba C, Amoros I, et al. Should Urine-LAM Tests Be Used in TB Symptomatic HIV-Positive Patients When No CD4 Count Is Available? A Prospective Observational Cohort Study From Malawi. J Acquir Immune Defic Syndr. 2020;83(1):24–30. Epub 2019/10/22. doi: 10.1097/QAI.0000000000002206 ; PubMed Central PMCID: PMC6903332. 31633613 PMC6903332

[31] Nakiyingi L, Moodley VM, Manabe YC, Nicol MP, Holshouser M, Armstrong DT, et al. Diagnostic accuracy of a rapid urine lipoarabinomannan test for tuberculosis in HIV-infected adults. Journal of acquired immune deficiency syndromes (1999). 2014;66(3):270–9. doi: 10.1097/QAI.0000000000000151 . 24675585 PMC4146703

[32] Bjerrum S, Schiller I, Dendukuri N, Eisenhut M, Kohli M, Nathavitharana RR. Web Annex A. LF-LAM for the diagnosis of active tuberculosis in people living with HIV: an updated systematic review. In: Lateral flow urine lipoarabinomannan assay (LF-LAM) for the diagnosis of active tuberculosis in people living with HIV: policy update (2019) Geneva: World Health Organization 2019.

