# Supplementary material for: Prospective multicentre accuracy evaluation of the FUJIFILM SILVAMP TB LAM test for the diagnosis of tuberculosis in people living with HIV demonstrates lot-to-lot variability
Source: PLoS One. 2024 May 31;19(5):e0303846. doi: 10.1371/journal.pone.0303846 (PMC11142480; doi:10.1371/journal.pone.0303846)
Supplement: S2 File — (DOCX) [file pone.0303846.s002.docx]

**S2. Supplementary methods and results**

**Supplementary methods**

***Procedures***

The 70 urine samples selected from the FIND biobank were representatives of both microbiologically confirmed TB and non-TB patients. All 70 samples had their LAM concentration previously determined by the EclLAM assay, thus they also served as control material for the post-hoc lot-to-lot assessment. A total of 47 of the 50 microbiologically confirmed TB samples had both sputum culture and smear positive results and three of the 50 TB positives were confirmed by Xpert MTB/RIF. Their selection mainly centred around the 10–50 pg/mL LAM concentration range, which includes the limit of detection (LoD) of the FujiLAM test (~ 30 pg/mL) as lot-to-lot variability is more likely to impact the threshold for LAM detection. The remaining 20 samples were TB-negative (as per MRS) with undetectable LAM concentration by the EclLAM assay (below the LoD of 11pg/mL), thus ideally should not yield a signal with the FujiLAM test.

***FujiLAM testing***

FujiLAM testing was done as per manufacturer’s instructions. Briefly, urine was added to the reagent tube up to the indicator line (approximately 200 µl), mixed, and incubated for 40 minutes at ambient temperature. After mixing again, two drops of urine/reagent are added to the test strip. Following this, a button is pressed immediately to release a reducing agent for silver amplification. After the colour indicator mark turns orange (approximately 10 minutes), the next button is pressed. The result is then read within 10 minutes. The FujiLAM assay does not use a reference scale card and any line seen on the test is considered positive.

Operators were trained prior to the study start and their competency was assessed using a proficiency testing tool (see page 3) composed of the following four sections:

1. FujiLAM test run on a blinded mock urine sample observed by a moderator
2. Appraisal of the moderator
3. Questionnaire to assess operator’s understanding of the FujiLAM test procedure
4. Test result interpretation using photographs of FujiLAM results.

***AlereLAM testing***

AlereLAM testing was done as per manufacturer’s instructions. Operators were trained prior to the study start and their competency was assessed using a proficiency testing tool (see page 11) composed of the following 4 sections:

1. AlereLAM test run on a blinded mock urine sample observed by a moderator
2. Appraisal of the moderator
3. Questionnaire to assess operator’s understanding of the AlereLAM test procedure
4. Test result interpretation using photographs of AlereLAM results.

Both FujiLAM and AlereLAM tests were done within 2 hours of sample collection at room temperature.

***Statistical analysis***

Variables such as age, sex, country, visit day, CD4 cell count and hospitalization were included as they have previously been shown to be related to the accuracy of diagnostic tests. Variables such as lot and reader were included to assess whether the variability was related to manufacturing or training procedures/readers’ performance. Additionally, urine characteristics were included to investigate whether the presence of particles or blood in the urine affected test outcome.

To investigate whether the sensitivity and specificity of FujiLAM differed significantly for the same reasons, we fitted GLMMs in TB positive and negative patients (per eMRS reference) separately.

The relationship between the CD4 counts and FujiLAM/eMRS result mismatch was further investigated by a linear regression, using the “lm” function in the base package. CD4 counts were categorized based on previous thresholds for visual representation. The linear model was constructed using the median CD4 value of each category.

**Supplementary results**

***Six-month follow-up***

The 6-month follow-up was undertaken to address the discrepant result between the FujiLAM index test and the reference standard (composite reference standard). A total of 125 patients (26 inpatients, 99 outpatients) with baseline FujiLAM-positive results (Day 1 and/or Day 2 urine) but negative composite reference standard results were eligible to come back at 6 months. In total, 116 (21 inpatients, 95 outpatients) out of the 125 patients attended the follow-up visit, while 9 patients (5 inpatients, 9 outpatients) did not come back at the 6-month visit (2 passed away, 7 were lost to follow-up). Sputum and urine samples were collected for 16 inpatients and for 41 outpatients if signs/symptoms had not improved or completely resolved. Urine samples were tested on FujiLAM, AlereLAM and Xpert Ultra and sputum samples were tested on smear, Löwenstein-Jensen medium, Mycobacteria Growth Indicator Tube and Xpert Ultra. Five of the 57 urine samples resulted in a positive FujiLAM and one of these five had positive AlereLAM test result as well. Four of the five FujiLAM positives were tested using lot 19003 and one was tested using lot 20003. All the available reference test results were negative, indicating that these patients were unlikely to have TB.
