# Supplementary material for: Prospective multicentre accuracy evaluation of the FUJIFILM SILVAMP TB LAM test for the diagnosis of tuberculosis in people living with HIV demonstrates lot-to-lot variability
Source: PLoS One. 2024 May 31;19(5):e0303846. doi: 10.1371/journal.pone.0303846 (PMC11142480; doi:10.1371/journal.pone.0303846)
Supplement: S3 File — (DOCX) [file pone.0303846.s003.docx]

**S3. FujiLAM proficiency testing tool**

**FUJIFILM SILVAMP TB LAM (FujiLAM) – PROFICIENCY TESTING TOOL**

**Intended use**

This tool is intended to be used by the moderator to assess the minimum training needs and the resulting proficiency of health care workers who are intended to be end users of the FUJIFILM SILVAMP TB LAM (FujiLAM) test. This proficiency assessment will be carried out after initial training. Once operators have processed at least two FujiLAM tests during the training, each operator will be asked to independently perform one complete FujiLAM test runs using blinded mock urine sample. The moderator will observe, without intervening or correcting mistakes.

The proficiency testing comprise of the following parts:

1. **Observed hands-on FujiLAM test run**. Each operator will be provided with one blinded mock urine sample and asked to perform the FujiLAM test. The moderator will observe the test run and complete a proficiency checklist to assess adherence to the critical steps according to the FujiLAM Quick Reference Guide/Instructions for Use (IFU)/video to ensure reliable test results.
2. **Appraisal**. At the end, the moderator will estimate the level of confidence shown by the operator while performing the FujiLAM tests.
3. **Questionnaire**. 10 questions will be asked orally by the moderator after the test run to assess the level of understanding of the FujiLAM test and the operators’ ability in coping with problems that might arise during the procedure.
4. **Test result interpretation**. Each operator will be provided with photographs of possible test results including positive, negative and invalid. Although the photographs are not the same as test results in real, this will allow the assessment of results interpretation of different band intensities.

**Performance targets**

The training will be considered successful if the following performance targets are met:

- Individual scores for Part A, C and D ≥ 22 (80%), ≥8/10 (80%) and ≥9 (90%), respectively
- **AND** an overall appraisal of the operator’s confidence to perform FujiLAM of ≥ 4 (on a scale of 1-5)

If performance targets are not met after initial training, the operator will undergo additional training on specific topics, and be reassessed for proficiency until targets are met.

**Materials needed**:

| **FujiLAM kit (materials included)** | **Materials not provided** |
| --- | --- |
| Pipette | Urine collection cup |
| Tube Rack | Gloves |
| Nozzle | Permanent marker |
| Quick Reference Guide | Biohazard waste bin |
| Instructions For Use | Artificial urine samples ( 10 ml of 5 ng/ml LAM in buffer as positive control and 10 ml of sterile water as negative control) |
| Test pouch (test cartridge & reagent tube) |  |

1. **Observed hands-on FujiLAM test run – Checklist**

**Instructions:**

- Prepare the workspace.
- Process 1 sample according to the FujiLAM Quick Reference Guide/Instructions for Use.
- Two moderators will be present: One of them will video record the session and the other will complete the checklist below.
- The operator has to perform the tasks outlined in the checklist correctly. If not, the answer should be “NO” and an explanation should be added on the last column.
- For each correctly performed item, the operator will obtain 1 point.

NAME OF OPERATOR: __________________________ DATE OF COMPLETION: _________________

| **Procedure** | **Step** | **Assessment of sample (If NO add comment)** | | **Comment** |
| --- | --- | --- | --- | --- |
| **FujiLAM preparation** | 1. Did the operator collect all necessary materials (the kit and additional materials needed including urine sample) as outlined in the instructions? | ❑YES | ❑NO |  |
|  | 1. Did the operator open the cardboard box correctly? | ❑YES | ❑NO |  |
|  | 1. Did the operator put on appropriate protective equipment (gloves, lab coat)? | ❑YES | ❑NO |  |
|  | 1. Did the operator check the expiry date on the test pouch? | ❑YES | ❑NO |  |
|  | 1. Did the operator open the test pouch | ❑YES | ❑NO |  |
|  | 1. Did the operator remove both test cartridge and reagent tube from the pouch? | ❑YES | ❑NO |  |
|  | 1. Did the operator write clearly and at the appropriate place the patient ID on the test cartridge and reagent tube? | ❑YES | ❑NO |  |
| **FujiLAM sample preparation** | 1. Did the operator visually inspect the reagent tube for presence of a pad? | ❑YES | ❑NO |  |
|  | 1. Did the operator remove the seal of the reagent tube? | ❑YES | ❑NO |  |
|  | 1. Did the operator open the urine container at a time and close lid afterwards? | ❑YES | ❑NO |  |
|  | 1. Did the operator transfer urine to the correct tube? | ❑YES | ❑NO |  |
|  | 1. Was the correct volume of urine transferred to the reagent tube (up to indicator line)? | ❑YES | ❑NO |  |
|  | 1. Did the operator correctly attach the nozzle to the tube? | ❑YES | ❑NO |  |
|  | 1. Was the tube mixed gently 10 times, without inverting the tube? | ❑YES | ❑NO |  |
|  | 1. Did the operator incubate the sample for 40-50 minutes at room temperature? | ❑YES | ❑NO |  |
|  | 1. Was the tube, after the incubation, mixed gently again 10 times without inverting the tube? | ❑YES | ❑NO |  |
| **FujiLAM test procedure** | 1. Did the operator hold the reagent tube at 90 degrees to the test device while adding the drops? | ❑YES | ❑NO |  |
|  | 1. Did the operator hold the tube at about 1 cm height while adding the drops? | ❑YES | ❑NO |  |
|  | 1. Was the correct amount of the sample mixture (2 drops) added to the well (1) on the test cartridge? | ❑YES | ❑NO |  |
|  | 1. Was button (2) pushed immediately and completely by the operator until it became dented? | ❑YES | ❑NO |  |
|  | 1. Did the operator wait until the “Go Next” mark turned orange? | ❑YES | ❑NO |  |
|  | 1. Did the operator proceed to the next step within 30 mins? | ❑YES | ❑NO |  |
|  | 1. Was button (3) pushed completely by the operator until it became dented? | ❑YES | ❑NO |  |
| **FujiLAM interpretation of results** | 1. Was the result read within 1-10 minutes after the control line appeared? | ❑YES | ❑NO |  |
|  | 1. Was the operator able to correctly interpret the test result? | ❑YES | ❑NO |  |
|  | 1. Was the operator able to correctly record the test results and any comments on the FujiLAM result form? | ❑YES | ❑NO |  |
|  | 1. Did the operator dispose all urine specimens and assay materials in the appropriate biohazard waste bin? | ❑YES | ❑NO |  |
|  | 1. Did the operator disinfect the bench after use? | ❑YES | ❑NO |  |
| **PART A** | **Score / Number of correct items** | **/ 28** | | …………… % |

NAME OF MODERATOR: __________________________ DATE REVIEWED:_______________________

1. **Appraisal**

- The moderator that supervises the assessment will estimate the level of confidence is shown by the operator while performing the FujiLAM test.

NAME OF OPERATOR: __________________________ DATE OF COMPLETION: _________________

| **APPRAISAL** | How would you evaluate the level of confidence shown by the operator while performing FujiLAM?  ❑1 (not confident) ❑2 ❑3 ❑4 ❑5 (very confident)  Comments: |
| --- | --- |

NAME OF MODERATOR: __________________________ DATE REVIEWED:_______________________

1. **Questionnaire**

**Instructions:**

- The moderator will ask 10 out of the 29 following questions to each operator (individually) in the context of the FujiLAM proficiency run.
- For each correct item, the operator will obtain 1 point.

NAME OF OPERATOR: __________________________ DATE OF COMPLETION:_________________

| **FujiLAM Questions** | | **Answered correctly** | | **If NO add comment** |
| --- | --- | --- | --- | --- |
| 1. What is the intended use of FujiLAM? | | ❑YES | ❑NO |  |
| 1. What is the storage temperature of the kit? | | ❑YES | ❑NO |  |
| 1. Can you describe all the components that are included in the FujiLAM kit? | | ❑YES | ❑NO |  |
| 1. What kind of specimen is required for the FujiLAM test? | | ❑YES | ❑NO |  |
| 1. What do you do if the test device was dropped prior to testing? | | ❑YES | ❑NO |  |
| 1. Which lighting condition should be avoided while using the cartridge? | | ❑YES | ❑NO |  |
| 1. What would you do if the pouch of the device was not opened right before testing but well in advance i.e. contents exposed to ambient conditions? | | ❑YES | ❑NO |  |
| 1. Which two things do you need to check (visually) before carrying out the FujiLAM test? | | ❑YES | ❑NO |  |
| 1. What would you do if the expiry date of the test has passed? | | ❑YES | ❑NO |  |
| 1. What would you do if you realize the device is broken? | | ❑YES | ❑NO |  |
| 1. What would you do if you do not observe a pad in the reagent tube? | | ❑YES | ❑NO |  |
| 1. What would you do if the amount of urine that you transferred to the reagent tube is above the indicator line? | | ❑YES | ❑NO |  |
| 1. How critical is the urine volume added to the reagent tube? | | ❑YES | ❑NO |  |
| 1. What precaution do you need to take when mixing the reagent tube after adding urine? | | ❑YES | ❑NO |  |
| 1. What is the minimum time the urine has to be incubated in the reagent tube? | | ❑YES | ❑NO |  |
| 1. What is the maximum time you can let the reagent tube be incubated? | | ❑YES | ❑NO |  |
| 1. What would you do if you dropped the reagent tube containing the sample mixture and it splashed on your hands, face and/or eyes? | | ❑YES | ❑NO |  |
| 1. What would you do if you dropped the reagent tube containing the sample mixture and it splashed on the FujiLAM cartridge? | | ❑YES | ❑NO |  |
| 1. How many drops of the sample mixture should be transferred to the FujiLAM cartridge? | | ❑YES | ❑NO |  |
| 1. What would you do if you observe bubbles in the sample well (1)? | | ❑YES | ❑NO |  |
| 1. What should you do straight after transferring 2 drops of the sample mixture to the FujiLAM cartridge? | | ❑YES | ❑NO |  |
| 1. What would you do, if you pressed button (2) and then realized that you added only 1 drop of sample mixture? | | ❑YES | ❑NO |  |
| 1. How long should you wait after pressing button (2)? | | ❑YES | ❑NO |  |
| 1. What would you do if the ‘Go Next’ mark does not appear after 30 mins? | | ❑YES | ❑NO |  |
| 1. What is the next step after the ‘Go Next’ mark turns orange? | | ❑YES | ❑NO |  |
| 1. What would you do if, you realized that you read the orange ‘Go Next’ mark more than 30 minutes after you pressed button (2)? | | ❑YES | ❑NO |  |
| 1. What would you do if the control band does not appear after approximately 1 minute? | | ❑YES | ❑NO |  |
| 1. What is the maximum time within which you need to interpret the test results? | | ❑YES | ❑NO |  |
| 1. How should you dispose of the FujiLAM cartridge? | | ❑YES | ❑NO |  |
| **PART C** | **Score / Number of correct questions** | **/ 10** | | …………… % |

NAME OF MODERATOR: __________________________ DATE REVIEWED:_________________

1. **Test result interpretation**

**Instructions:**

- The moderator will provide a form containing 10 photographs of possible test results.
- The operator has to interpret the result for each case.
- For each correct item, the operator will obtain 1 point.

NAME OF OPERATOR: __________________________ DATE OF COMPLETION:_________________

|  | **Test result example** | **Result interpretation** | | | **Moderator’s comment** |
| --- | --- | --- | --- | --- | --- |
| 1 | 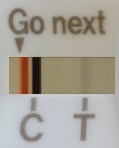 | ❑Positive | ❑Negative | ❑Invalid |  |
| 2 | 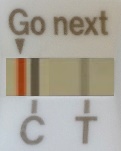 | ❑Positive | ❑Negative | ❑Invalid |  |
| 3 | 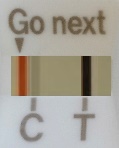 | ❑Positive | ❑Negative | ❑Invalid |  |
| 4 | 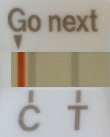 | ❑Positive | ❑Negative | ❑Invalid |  |
| 5 | 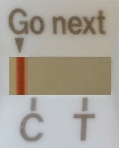 | ❑Positive | ❑Negative | ❑Invalid |  |
| 6 | 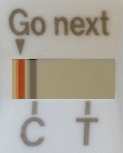 | ❑Positive | ❑Negative | ❑Invalid |  |
| 7 | 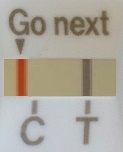 | ❑Positive | ❑Negative | ❑Invalid |  |
| 8 | 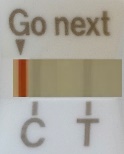 | ❑Positive | ❑Negative | ❑Invalid |  |
| 9 | 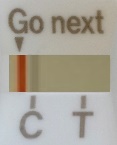 | ❑Positive | ❑Negative | ❑Invalid |  |
| 10 | 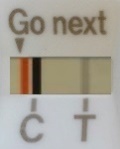 | ❑Positive | ❑Negative | ❑Invalid |  |
| **PART D** | | **Score / Number of correct items** | | **/ 10** | …………… % |

1. **Conclusion**

| **Performance targets met?** |  |  | **If NO add comment** |
| --- | --- | --- | --- |
| Score Part A: ≥80%? | ❑YES | ❑NO |  |
| Score Part B: Appraisal ≥4? | ❑YES | ❑NO |  |
| Score Part C: ≥80%? | ❑YES | ❑NO |  |
| Score Part D: ≥90%? | ❑YES | ❑NO |  |
| **Conclusion: Operator passed proficiency test** | **❑YES**^#^ | **❑NO** |  |

^#^Operator can only pass the proficiency test, when score for individual Parts A, B, C and D were ALL met.

NAME OF MODERATOR: __________________________ DATE ASSESSED:_________________
