# Supplementary material for: Prospective multicentre accuracy evaluation of the FUJIFILM SILVAMP TB LAM test for the diagnosis of tuberculosis in people living with HIV demonstrates lot-to-lot variability
Source: PLoS One. 2024 May 31;19(5):e0303846. doi: 10.1371/journal.pone.0303846 (PMC11142480; doi:10.1371/journal.pone.0303846)
Supplement: S4 File — (DOCX) [file pone.0303846.s004.docx]

**S4. AlereLAM proficiency testing tool**

**ALERE DETERMINE™ TB LAM (AlereLAM) – PROFICIENCY TESTING TOOL**

**Intended use**

This tool is intended to be used by the moderator to assess the minimum training needs and the resulting proficiency of health care workers who are intended to be end users of the ALERE DETERMINE™ TB LAM (AlereLAM) test. This proficiency assessment will be carried out after initial training. Once operators have processed at least two AlereLAM tests during the training, each operator will be asked to independently perform one complete AlereLAM test run using blinded mock urine sample. The moderator will observe, without intervening or correcting mistakes.

The proficiency testing comprise of the following parts:

1. **Observed hands-on AlereLAM test run**. Each operator will be provided with one blinded mock urine sample and asked to perform the AlereLAM test. The moderator will observe the test run and complete a proficiency checklist to assess adherence to the critical steps according to the AlereLAM Quick Reference Guide/Instructions for Use (IFU) to ensure reliable test results.
2. **Appraisal**. At the end, the moderator will estimate the level of confidence shown by the operator while performing the AlereLAM tests.
3. **Questionnaire**. 5 questions out of the 13 will be asked orally by the moderator after the test run to assess the level of understanding of the AlereLAM test and the operators’ ability in coping with problems that might arise during the procedure.
4. **Test result interpretation**. Each operator will be provided with photographs of possible test results including positive, negative and invalid. Although the photographs are not the same as test results in real, this will allow the assessment of results interpretation of different band intensities. Use the reference card for the interpretation.

**Performance targets**

The training will be considered successful if the following performance targets are met:

- Individual scores for Part A, C and D ≥ 22 (80%), ≥ 4 (80%) and ≥9 (90%), respectively
- **AND** an overall appraisal of the operator’s confidence to perform AlereLAM of ≥ 4 (on a scale of 1-5)

If performance targets are not met after initial training, the operator will undergo additional training on specific topics, and be reassessed for proficiency until targets are met.

**Materials needed**:

| **AlereLAM kit (materials included)** | **Materials not provided** |
| --- | --- |
| Instructions For Use | Urine collection cup |
| Test | Gloves |
| Reference Card | Permanent marker |
|  | Pipette |
|  | Biohazard waste bin |
|  | Artificial urine samples ( 10 ml of 5 ng/ml LAM in buffer as positive control and 10 ml of sterile water as negative control) |
|  |  |

1. **Observed hands-on FujiLAM test run – Checklist**

**Instructions:**

- Prepare the workspace.
- Process 1 sample according to the AlereLAM Instructions for Use.
- The moderator will complete the checklist below.
- The operator has to perform the tasks outlined in the checklist correctly. If not, the answer should be “NO” and an explanation should be added on the last column.
- For each correctly performed item, the operator will obtain 1 point.

NAME OF OPERATOR: __________________________ DATE OF COMPLETION: _________________

| **Procedure** | **Step** | **Assessment of sample (If NO add comment)** | | **Comment** |
| --- | --- | --- | --- | --- |
| **AlereLAM preparation** | 1. Did the operator collect all necessary materials (the kit and additional materials needed including urine sample) as outlined in the instructions? | ❑YES | ❑NO |  |
|  | 1. Did the operator open the aluminium pouch correctly? | ❑YES | ❑NO |  |
|  | 1. Did the operator put on appropriate protective equipment (gloves, lab coat)? | ❑YES | ❑NO |  |
|  | 1. Did the operator check the expiry date on the test pouch? | ❑YES | ❑NO |  |
|  | 1. Did the operator open the test tearing at the perforation? | ❑YES | ❑NO |  |
|  | 1. Did the operator remove the protecting foil cover completely? | ❑YES | ❑NO |  |
|  | 1. Did the operator write clearly and at the appropriate place the patient ID on the test strip? | ❑YES | ❑NO |  |
| **AlereLAM test procedure** | 1. Did the operator transfer 60 ul urine to the correct position of the test strip? | ❑YES | ❑NO |  |
|  | 1. Did the operator incubate the test in horizontal position? | ❑YES | ❑NO |  |
|  | 1. Did the operator wait 25 minutes before interpreting the results? | ❑YES | ❑NO |  |
|  | 1. Did the operator interpret the results within 35 minutes? | ❑YES | ❑NO |  |

| **AlereLAM interpretation of results** | 1. Was the operator able to correctly interpret the test result? | ❑YES | ❑NO |  |
| --- | --- | --- | --- | --- |
|  | 1. Was the operator able to correctly record the test results and any comments on the AlereLAM result form? | ❑YES | ❑NO |  |
|  | 1. Did the operator dispose all urine specimens and assay materials in the appropriate biohazard waste bin? | ❑YES | ❑NO |  |
|  | 1. Did the operator disinfect the bench after use? | ❑YES | ❑NO |  |
| **PART A** | **Score / Number of correct items** | **/ 15** | …………… % |  |

NAME OF MODERATOR: __________________________ DATE REVIEWED:_______________________

1. **Appraisal**

- The moderator that supervises the assessment will estimate the level of confidence is shown by the operator while performing the AlereLAM test.

NAME OF OPERATOR: __________________________ DATE OF COMPLETION: _________________

| **APPRAISAL** | How would you evaluate the level of confidence shown by the operator while performing AlereLAM?  ❑1 (not confident) ❑2 ❑3 ❑4 ❑5 (very confident)  Comments: |
| --- | --- |

NAME OF MODERATOR: __________________________ DATE REVIEWED:_______________________

1. **Questionnaire**

**Instructions:**

- The moderator will ask 5 out of the 13 following questions to each operator (individually) in the context of the AlereLAM proficiency run.
- For each correct item, the operator will obtain 1 point.

NAME OF OPERATOR: __________________________ DATE OF COMPLETION:_________________

| **AlereLAM Questions** | | **Answered correctly** | | **If NO add comment** |
| --- | --- | --- | --- | --- |
| 1. What is the intended use of AlereLAM? | | ❑YES | ❑NO |  |
| 1. What is the storage temperature of the test? | | ❑YES | ❑NO |  |
| 1. Can you describe all the components that are included in the AlereLAM pouch? | | ❑YES | ❑NO |  |
| 1. What kind of specimen is required for the AlereLAM test? | | ❑YES | ❑NO |  |
| 1. Which lighting condition should be avoided while using the test? | | ❑YES | ❑NO |  |
| 1. What would you do if the pouch of the device was not opened right before testing but more than 2 hours in advance i.e. contents exposed to ambient conditions? | | ❑YES | ❑NO |  |
| 1. What would you do if the expiry date of the test has passed? | | ❑YES | ❑NO |  |
| 1. What would you do if you realize the lot number on the pouch is not identical to the one on the test strips? | | ❑YES | ❑NO |  |
| 1. What would you do if the amount of urine that you transferred to the test is more than 60 ul? | | ❑YES | ❑NO |  |
| 1. What would you do if you dropped the urine container and it splashed on the AlereLAM test(s)? | | ❑YES | ❑NO |  |
| 1. What is the minimum time to wait before test result interpretation? | | ❑YES | ❑NO |  |
| 1. What is the maximum time within which you need to interpret the test results? | | ❑YES | ❑NO |  |
| 1. How should you dispose of the AlereLAM cartridge? | | ❑YES | ❑NO |  |
| **PART C** | **Score / Number of correct questions** | **/ 5** | | …………… % |

NAME OF MODERATOR: __________________________ DATE REVIEWED:_________________

1. **Test result interpretation**

**Instructions:**

- The moderator will provide a form containing 10 photographs of possible test results.
- The operator has to interpret the result for each case using the reference card of the AlereLAM test.
- For each correct item, the operator will obtain 1 point.

NAME OF OPERATOR: __________________________ DATE OF COMPLETION:_________________

|  | **Test result example** | **Result interpretation** | | | **Moderator’s comment** |
| --- | --- | --- | --- | --- | --- |
| 1 | 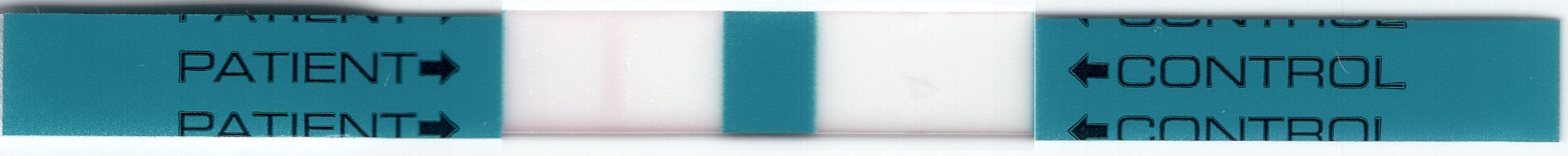 | ❑Positive  ❑1+  ❑2+  ❑3+  ❑4+ | ❑Negative | ❑Invalid |  |
| 2 | 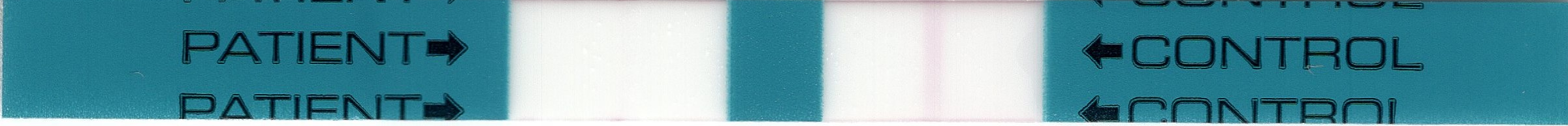 | ❑Positive  ❑1+  ❑2+  ❑3+  ❑4+ | ❑Negative | ❑Invalid |  |
| 3 | 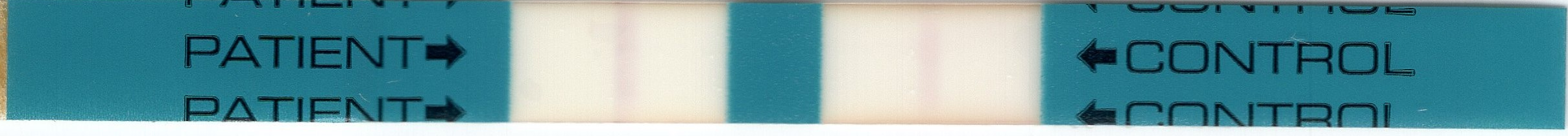 | ❑Positive  ❑1+  ❑2+  ❑3+  ❑4+ | ❑Negative | ❑Invalid |  |
| 4 | 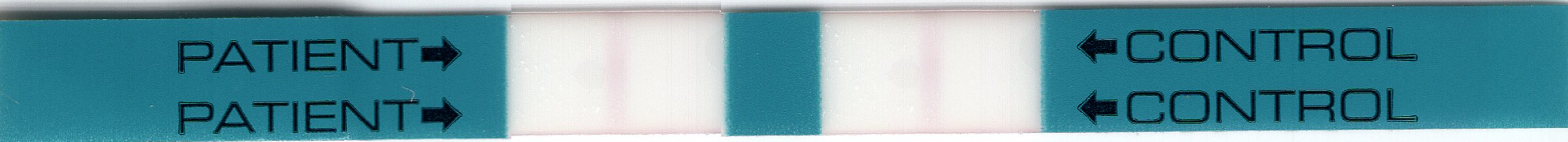 | ❑Positive  ❑1+  ❑2+  ❑3+  ❑4+ | ❑Negative | ❑Invalid |  |
| 5 | 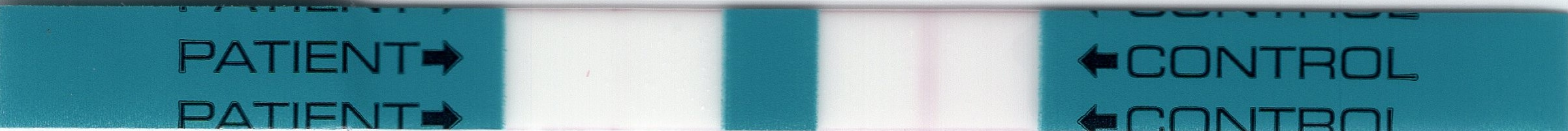 | ❑Positive  ❑1+  ❑2+  ❑3+  ❑4+ | ❑Negative | ❑Invalid |  |
| 6 | 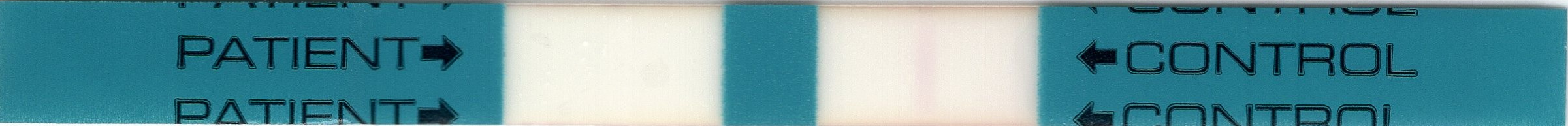 | ❑Positive  ❑1+  ❑2+  ❑3+  ❑4+ | ❑Negative | ❑Invalid |  |
| 7 | 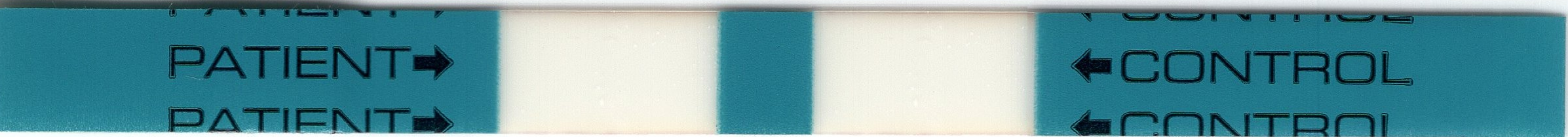 | ❑Positive  ❑1+  ❑2+  ❑3+  ❑4+ | ❑Negative | ❑Invalid |  |
| 8 | 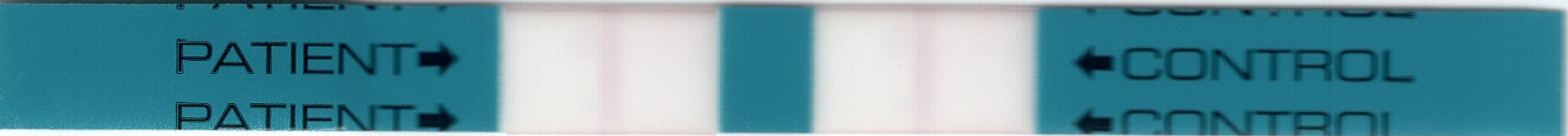 | ❑Positive  ❑1+  ❑2+  ❑3+  ❑4+ | ❑Negative | ❑Invalid |  |
| 9 | 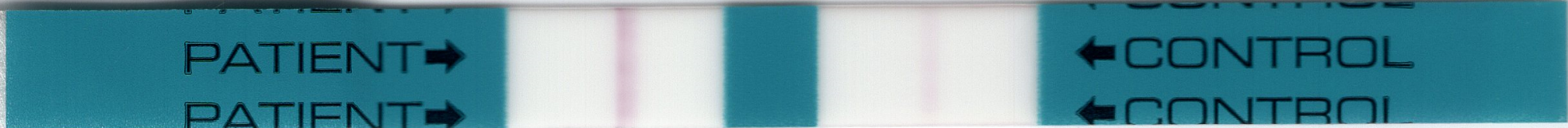 | ❑Positive  ❑1+  ❑2+  ❑3+  ❑4+ | ❑Negative | ❑Invalid |  |
| 10 | 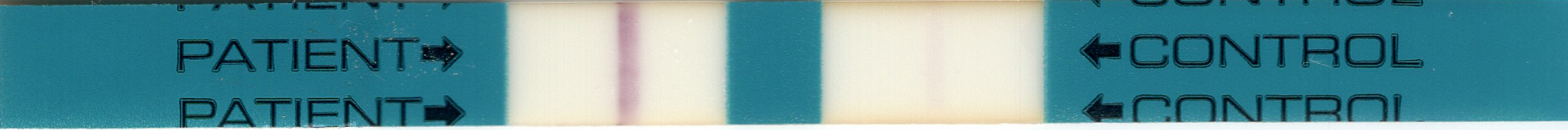 | ❑Positive  ❑1+  ❑2+  ❑3+  ❑4+ | ❑Negative | ❑Invalid |  |
| **PART D** | | **Score / Number of correct items** | | **/ 10** | …………… % |

1. **Conclusion**

| **Performance targets met?** |  |  | **If NO add comment** |
| --- | --- | --- | --- |
| Score Part A: ≥80%? (≥12) | ❑YES | ❑NO |  |
| Score Part B: Appraisal ≥4? | ❑YES | ❑NO |  |
| Score Part C: ≥80%? (≥10.4) | ❑YES | ❑NO |  |
| Score Part D: ≥90%? | ❑YES | ❑NO |  |
| **Conclusion: Operator passed proficiency test** | **❑YES**^#^ | **❑NO** |  |

^#^Operator can only pass the proficiency test, when score for individual Parts A, B, C and D were ALL met.

NAME OF MODERATOR: __________________________ DATE ASSESSED:_________________
