# Supplementary material for: Prospective multicentre accuracy evaluation of the FUJIFILM SILVAMP TB LAM test for the diagnosis of tuberculosis in people living with HIV demonstrates lot-to-lot variability
Source: PLoS One. 2024 May 31;19(5):e0303846. doi: 10.1371/journal.pone.0303846 (PMC11142480; doi:10.1371/journal.pone.0303846)
Supplement: S5 File — (DOCX) [file pone.0303846.s005.docx]

**S5. Figures and Tables**

**Figure 1. Study testing procedures**

1. **Day 1**


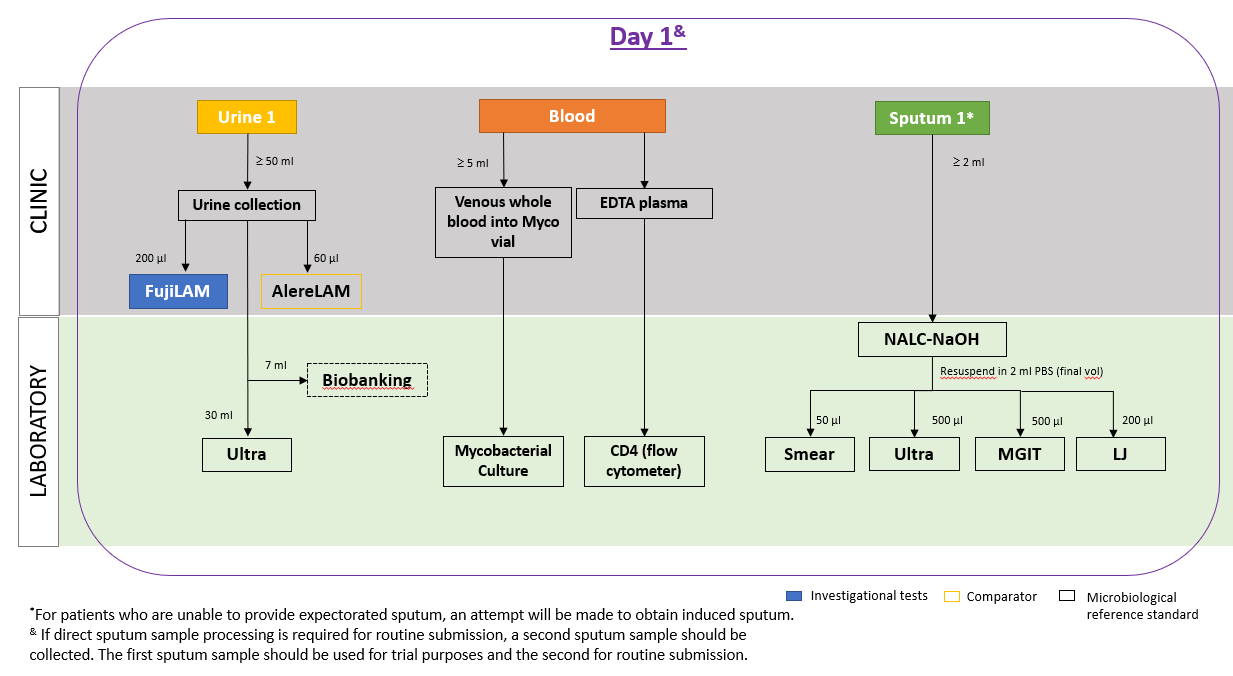


**B. Day 2**


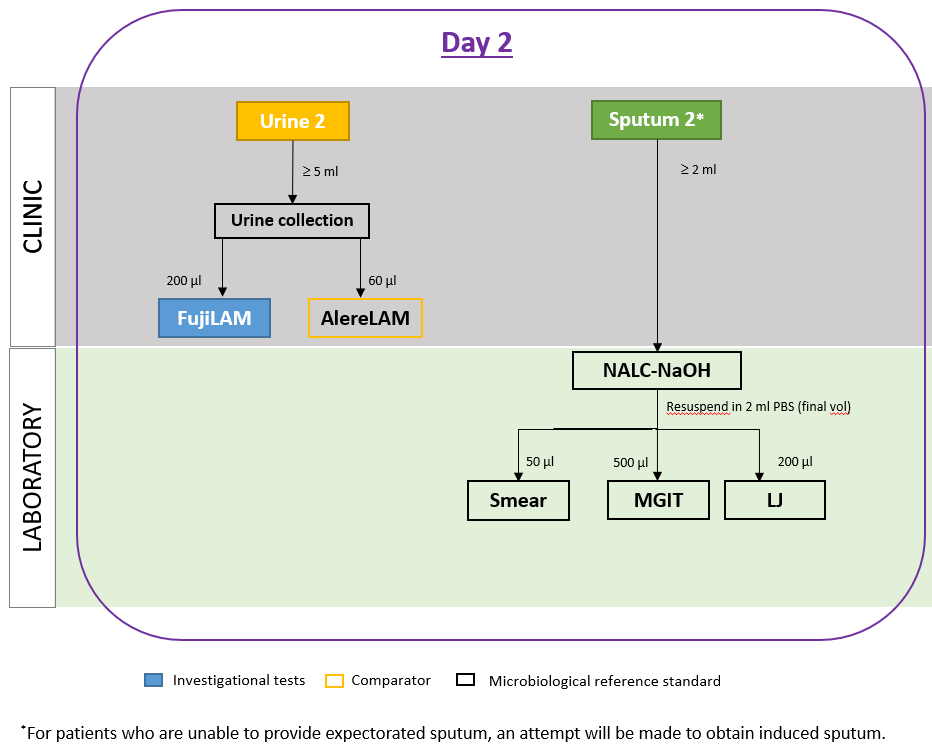


1. **
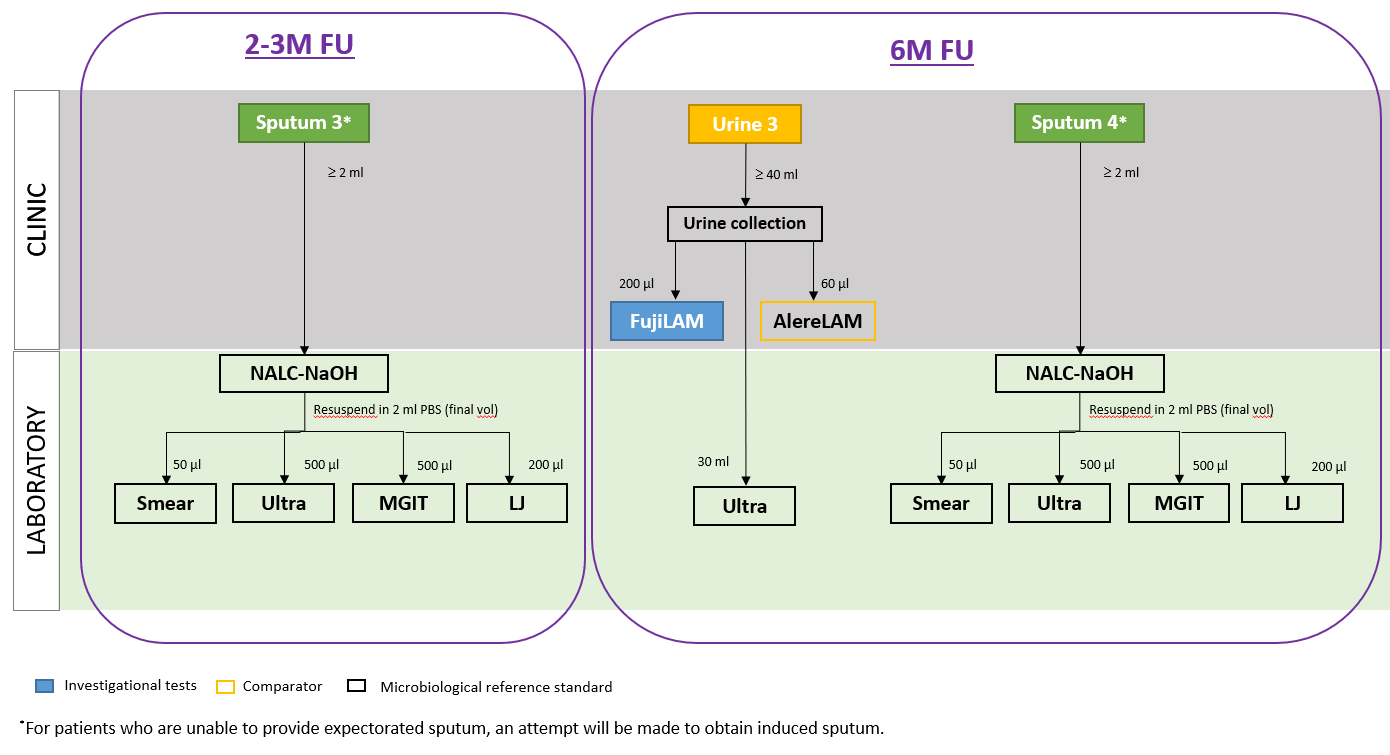
Follow-up**

LJ, Löwenstein-Jensen; MGIT, Mycobacteria Growth Indicator Tube.

**Figure 2: Lot distribution across countries**

**
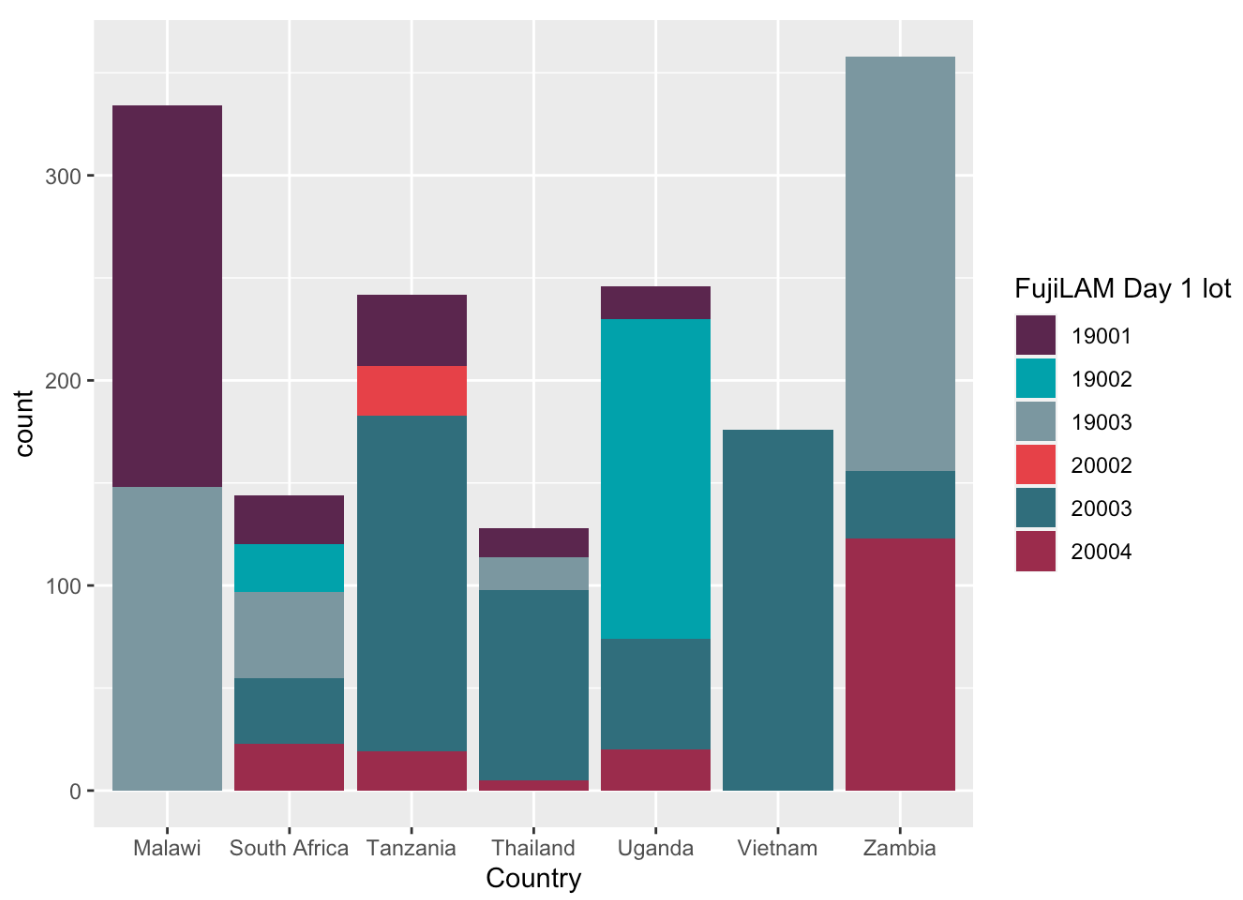
**

**Figure 3: Correlation of mismatch with CD4 cell count of TB positive cases**

**
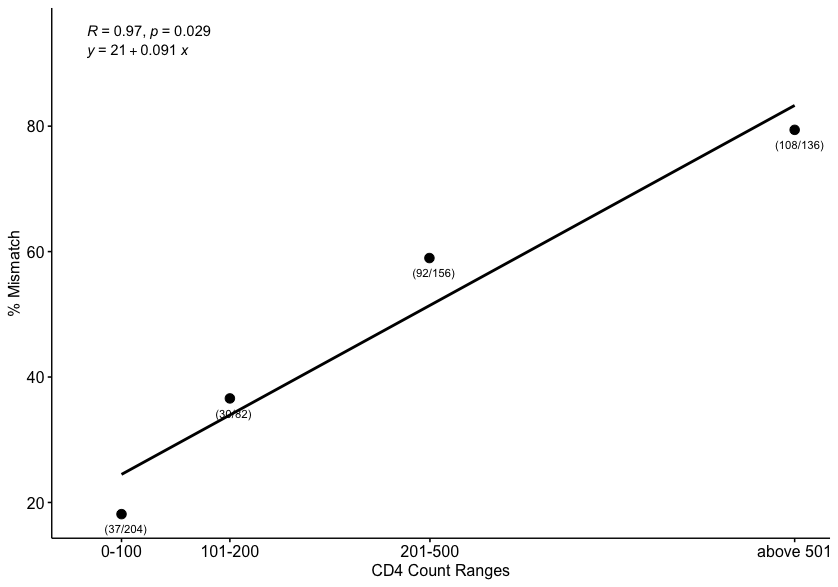
**

**Table 1. Participating centres, locations, settings, collection sites, IRB/IEC**

| **Country** | **Organization** | **Setting** | **Collection sites/City** | **Level of care** | **IRB/IEC** |
| --- | --- | --- | --- | --- | --- |
| **South Africa** | University of Cape Town | Inpatient | Mitchells Plain Hospital, Cape Town | District | University of Cape Town Faculty of Health Sciences Human Research Ethics Committee |
| **Malawi** | Malawi-Liverpool-Wellcome Programme and Kamuzu University of Health Sciences, Malawi (formerly College of Medicine, University of Malawi) | In- and outpatient | Queen Elizabeth Central Hospital, Blantyre | Regional referral | College of Medicine Research and Ethics Committee (COMREC), Kamuzu University of Health Sciences, Malawi |
|  |  |  | Bangwe Primary Care Clinic, Blantyre | Primary |  |
| **Zambia** | Centre for Infectious Disease Research in Zambia | In- and outpatient | Kanyama Hospital, Lusaka | Primary | University of Zambia Biomedical Research Ethics Committee,  National Health Research Authority |
|  |  |  | Chawama Hospital, Lusaka | Primary |  |
| **Uganda** | Infectious Diseases Institute | In- and outpatient | Kisenyi Health center IV Kampala | Sub-district | Infectious Disease Institute Scientific Review Committee,  Mulago Hospital Research and Ethics Committee,  Uganda National Council for Science and Technology |
|  |  |  | Mulago National Referral Hospital_Kiruddu, Kampala | National Referral |  |
| **Tanzania** | Swiss TPH & Ifakara Health Institute | Outpatient | Temeke Regional Referral Hospital, Dar es Salaam | Regional Referral | Ifakara Health Institute Review Board,  National Institute for Medical Research,  Tanzania Medicines and Medical Devices Authority |
| **Viet Nam** | Viet Tiep Hospital/National Lung Hospital | In- and outpatient | Viet Tiep Hospital, Hai Phong | Primary | National Lung Hospital Ethics Committee,  Ministry of Health |
|  |  |  |  |  |  |
| **Thailand** | HIV-NAT, Thai Red Cross AIDS Research Centre and Center of Excellence in Tuberculosis | In- and outpatient | Chulalongkorn Hospital, Bangkok | Referral | Institutional Review Board Faculty of Medicine of Chulalongkorn University |
|  |  |  | Bamrasnaradura Institute, Nothaburi | Referral | Institutional Review Board of Bamrasnaradura Infectious Disease Institute |
|  |  |  | Taksin Hospital, Bangkok | Referral | Bangkok Metropolitan Administration Human Research Ethics Committee |
|  |  |  | Public Health Center 28, Bangkok | Referral |  |

**IEC, independent ethics committee; IRC, Institutional Review Board.**

**Table 2. Number of urine samples per country selected for the post-hoc exploratory analysis and respective lots used in the prospective evaluation study**

| **Lot** | **19001** | **19002** | **19003** | **20002** | **20003** | **20004** | **Total (selected)** |
| --- | --- | --- | --- | --- | --- | --- | --- |
| **Malawi** | 16 | - | 1 | - | - | - | 17 |
| **Tanzania** | 15 | - | - | 2 | 3 | - | 20 |
| **Thailand** | 11 | - | 8 | - | - | - | 19 |
| **Zambia** | - | - | 38* | - | 4 | 14 | 55 |
| **Total** | 40 | 0 | 49 | 2 | 7 | 14 | 111 |

*One sample not tested on EclLAM due to insufficient volume.

**Table 3. Demographic and clinical characteristics of study participants stratified by country**

|  | **South Africa** | **Malawi** | **Zambia** | **Uganda** | **Tanzania** | **Viet Nam** | **Thailand** |
| --- | --- | --- | --- | --- | --- | --- | --- |
| N | 148 | 336 | 358 | 246 | 242 | 177 | 130 |
| Age median [min-max] (years) | 39 [21-64] | 40 [18-82] | 39 [18-70] | 39 [18-71] | 45 [18-75] | 43 [18-75] | 34 [18-70] |
| Female, no. (%) | 86/148 (58) | 188/336 (56) | 196/358 (55) | 144/246 (59) | 177/242 (73) | 37/177 (21) | 24/130 (18) |
| Median CD4 count - cells/µl [min-max] | 103.5 [0-1283] | 409 [7-1170] | 343 [3-1173] | 369 [2-3643] | 611 [18-1464] | 458 [10-1387] | 186 [0-1100] |
| Seriously ill*, no. (%) | 148/148 (100) | 7/336 (2) | 20/358 (6) | 28/246 (11) | 0/242 (0) | 13/177 (7) | 1/130 (1) |
| History of TB, no. (%) | 76/148 (51) | 62/336 (18) | 125/358 (35) | 39/246 (16) | 66/242 (27) | 52/177 (29) | 6/130 (5) |
| WHO TB Symptoms, no. (%) | 125/148 (84) | 312/336 (93) | 323/358 (90) | 239/246 (97) | 242/242 (100) | 156/177 (88) | 124/130 (95) |
| ***Setting*** |  |  |  |  |  |  |  |
| Inpatients, no. (%) | 148/148 (100) | 161/336 (48) | 141/358 (39) | 125/246 (51) | 0/242 (0) | 78/177 (44) | 31/130 (24) |
| Outpatients, no. (%) | 0/148 (0) | 175/336 (52) | 217/358 (61) | 121/246 (49) | 242/242 (100) | 99/177 (56) | 99/130 (76) |
| ***CD4*** |  |  |  |  |  |  |  |
| ≤100 | 71/148 (48) | 54/336 (16) | 53/358 (15) | 67/246 (27) | 15/242 (6) | 34/177 (19) | 41/130 (32) |
| >100 to ≤200 | 22/148 (15) | 43/336 (13) | 55/358 (15) | 17/246 (7) | 13/242 (5) | 9/177 (5) | 27/130 (21) |
| >200 to ≤500 | 28/148 (19) | 109/336 (32) | 143/358 (40) | 64/246 (26) | 59/242 (24) | 57/177 (32) | 48/130 (37) |
| >500 | 23/148 (16) | 129/336 (38) | 102/358 (28) | 97/246 (39) | 154/242 (64) | 75/177 (42) | 12/130 (9) |
| CD4 Unknown | 4/148 (3) | 1/336 (0) | 5/358 (1) | 1/246 (0) | 1/242 (0) | 2/177 (1) | 2/130 (2) |
| Seriously ill* - CD4 ≤100, no. (%) | 71/71 (100) | 1/54 (2) | 8/53 (15) | 11/67 (16) | 0/15 (0) | 11/34 (32) | 0/36 (0) |
| Seriously ill* - CD4 ≤200, no. (%) | 93/93 (100) | 3/97 (3) | 11/108 (10) | 13/84 (15) | 0/29 (0) | 11/43 (26) | 0/61 (0) |
| ***HIV treatment*** |  |  |  |  |  |  |  |
| ART interrupted, no. (%) | 42/148 (28) | 6/336 (2) | 2/358 (1) | 11/246 (4) | 5/242 (2) | 14/177 (8) | 10/130 (8) |
| Currently on ART, no. (%) | 68/148 (46) | 302/336 (90) | 315/358 (88) | 177/246 (72) | 209/242 (86) | 147/177 (83) | 54/130 (42) |
| Don t know, no. (%) | 0/148 (0) | 0/336 (0) | 9/358 (3) | 3/246 (1) | 0/242 (0) | 0/177 (0) | 6/130 (5) |
| Never used, no. (%) | 38/148 (26) | 28/336 (8) | 32/358 (9) | 55/246 (22) | 28/242 (12) | 16/177 (9) | 60/130 (46) |
| ***Speciation*** |  |  |  |  |  |  |  |
| NTM, no. (%) | 1/148 (1) | 0/336 (0) | 14/358 (4) | 12/246 (5) | 65/242 (27) | 0/177 (0) | 10/130 (8) |
| NTM&MTBC, no. (%) | 0/148 (0) | 1/336 (0) | 2/358 (1) | 0/246 (0) | 3/242 (1) | 0/177 (0) | 0/130 (0) |
| MTBC, no. (%) | 19/148 (13) | 19/336 (6) | 40/358 (11) | 30/246 (12) | 16/242 (7) | 14/177 (8) | 7/130 (5) |
| Not done/contaminated | 128/148 (86) | 316/336 (94) | 302/358 (84) | 204/246 (83) | 158/242 (65) | 163/177 (92) | 113/130 (87) |
| ***Follow-up status*** |  |  |  |  |  |  |  |
| Died within 3 months, no. (%) | 21/148 (14) | 27/336 (8) | 32/358 (9) | 37/246 (15) | 0/242 (0) | 4/177 (2) | 5/130 (4) |
| Alive, no. (%) | 70/148 (47) | 277/336 (82) | 256/358 (72) | 161/246 (65) | 241/242 (100) | 132/177 (75) | 113/130 (87) |
| Lost to follow-up, no. (%) | 17/148 (11) | 32/336 (10) | 33/358 (9) | 16/246 (7) | 1/242 (0) | 13/177 (7) | 12/130 (9) |
| No follow-up needed, no. (%) | 40/148 (27) | 0/336 (0) | 37/358 (10) | 32/246 (13) | 0/242 (0) | 28/177 (16) | 0/130 (0) |

*Seriously ill if any of the followings present: respiratory rate > 30 breaths/min, heart rate > 120 beats/min, body mass index [BMI] ≤ 18.5 kg/m2, systolic blood pressure < 90 mmHg or being unable to walk unaided

ART, antiretroviral therapy; FU, follow-up; MTBC, Mycobacterium tuberculosis complex; no. number; NTM, non-tuberculous mycobacteria; TB, tuberculosis; WHO, World Health Organization.

**Table 4. Sensitivity and specificity of Day 2 FujiLAM against the eMRS**

|  | **N** | **TP** | **FP** | **FN** | **TN** | **Sensitivity [95%CI]** | **Specificity [95%CI]** |
| --- | --- | --- | --- | --- | --- | --- | --- |
| All | 1615 | 149 | 241 | 143 | 1082 | 51.0 [45.3-56.7] | 81.8 [79.6-83.8] |
| ***CD4*** |  | | | | | | |
| ≤100 | 327 | 80 | 58 | 19 | 170 | 80.8 [72.0-87.4] | 74.6 [68.5-79.8] |
| 101 to ≤200 | 185 | 25 | 28 | 15 | 117 | 62.5 [47.0-75.8] | 80.7 [73.5-86.3] |
| 201 to ≤500 | 503 | 29 | 80 | 49 | 345 | 37.2 [27.3-48.3] | 81.2 [77.2-84.6] |
| >500 | 587 | 13 | 74 | 57 | 443 | 18.6 [11.2-29.2] | 85.7 [82.4-88.4] |
| Unknown | 13 | 2 | 1 | 3 | 7 | 40.0 [11.8-76.9] | 87.5 [52.9-97.8] |
| ***Setting*** |  | | | | | | |
| Inpatient | 671 | 92 | 85 | 50 | 444 | 64.8 [56.6-72.2] | 83.9 [80.6-86.8] |
| Outpatient | 944 | 57 | 156 | 93 | 638 | 38.0 [30.6-46.0] | 80.4 [77.4-83.0] |
| ***Country*** |  | | | | | | |
| South Africa | 142 | 41 | 31 | 14 | 56 | 74.5 [61.7-84.2] | 64.4 [53.9-73.6] |
| Malawi | 333 | 21 | 58 | 11 | 243 | 65.6 [48.3-79.6] | 80.7 [75.9-84.8] |
| Zambia | 351 | 29 | 62 | 23 | 237 | 55.8 [42.3-68.4] | 79.3 [74.3-83.5] |
| Uganda | 246 | 28 | 44 | 16 | 158 | 63.6 [48.9-76.2] | 78.2 [72.0-83.4] |
| Tanzania | 241 | 14 | 20 | 54 | 153 | 20.6 [12.7-31.6] | 88.4 [82.8-92.4] |
| Vietnam | 175 | 11 | 2 | 22 | 140 | 33.3 [19.8-50.4] | 98.6 [95.0-99.6] |
| Thailand | 127 | 5 | 24 | 3 | 95 | 62.5 [30.6-86.3] | 79.8 [71.7-86.1] |

eMRS, extended microbiological reference standard; FN, false negative; FP, false positive; N, number; TN, true negative; true positive.

**Table 5. Sensitivity and specificity of Day 1 and Day 2 AlereLAM against eMRS**

| **Day 1** | **N** | **TP** | **FP** | **FN** | **TN** | **Sensitivity [95%CI]** | **Specificity [95%CI]** |
| --- | --- | --- | --- | --- | --- | --- | --- |
| All | 1629 | 89 | 124 | 205 | 1211 | 30.3 [25.3-35.8] | 90.7 [89.0-92.2] |
| ***CD4*** |  | | | | | |  |
| ≤100 | 332 | 55 | 36 | 46 | 195 | 54.5 [44.8-63.8] | 84.4 [79.2-88.5] |
| 101 to ≤200 | 184 | 14 | 15 | 26 | 129 | 35.0 [22.1-50.5] | 89.6 [83.5-93.6] |
| 201 to ≤500 | 506 | 13 | 33 | 65 | 395 | 16.7 [10.0-26.5] | 92.3 [89.4-94.5] |
| >500 | 591 | 6 | 39 | 64 | 482 | 8.6 [4.0-17.5] | 92.5 [89.9-94.5] |
| Unknown | 16 | 1 | 1 | 4 | 10 | 20.0 [3.6-62.5] | 90.9 [62.3-98.4] |
| ***Setting*** |  | | | | | |  |
| Inpatient | 678 | 60 | 66 | 84 | 468 | 41.7 [33.9-49.8] | 87.6 [84.6-90.2] |
| Outpatient | 951 | 29 | 58 | 121 | 743 | 19.3 [13.8-26.4] | 92.8 [90.8-94.4] |
| ***Country*** |  | | | | | |  |
| South Africa | 144 | 17 | 1 | 39 | 87 | 30.4 [19.9-43.3] | 98.9 [93.8-99.8] |
| Malawi | 335 | 14 | 22 | 18 | 281 | 43.8 [28.2-60.7] | 92.7 [89.2-95.2] |
| Zambia | 358 | 12 | 46 | 41 | 259 | 22.6 [13.5-35.5] | 84.9 [80.5-88.5] |
| Uganda | 246 | 23 | 28 | 21 | 174 | 52.3 [37.9-66.2] | 86.1 [80.7-90.2] |
| Tanzania | 242 | 13 | 8 | 55 | 166 | 19.1 [11.5-30.0] | 95.4 [91.2-97.7] |
| Vietnam | 177 | 7 | 10 | 26 | 134 | 21.2 [10.7-37.8] | 93.1 [87.7-96.2] |
| Thailand | 127 | 3 | 9 | 5 | 110 | 37.5 [13.7-69.4] | 92.4 [86.2-96.0] |
| **Day 2** | **N** | **TP** | **FP** | **FN** | **TN** | **Sensitivity [95%CI]** | **Specificity [95%CI]** |
| All | 1616 | 82 | 165 | 209 | 1160 | 28.2 [23.3-33.6] | 87.5 [85.7-89.2] |
| ***CD4*** |  | | | | | |  |
| ≤100 | 328 | 52 | 36 | 47 | 193 | 52.5 [42.8-62.1] | 84.3 [79.0-88.4] |
| 101 to ≤200 | 183 | 11 | 16 | 29 | 127 | 27.5 [16.1-42.8] | 88.8 [82.6-93.0] |
| 201 to ≤500 | 505 | 10 | 56 | 67 | 372 | 13.0 [7.2-22.3] | 86.9 [83.4-89.8] |
| >500 | 587 | 7 | 55 | 63 | 462 | 10.0 [4.9-19.2] | 89.4 [86.4-91.7] |
| Unknown | 13 | 2 | 2 | 3 | 6 | 40.0 [11.8-76.9] | 75.0 [40.9-92.8] |
| ***Setting*** |  | | | | | |  |
| Inpatient | 672 | 58 | 62 | 83 | 469 | 41.1 [33.4-49.4] | 88.3 [85.3-90.8] |
| Outpatient | 944 | 24 | 103 | 126 | 691 | 16.0 [11.0-22.7] | 87.0 [84.5-89.2] |
| ***Country*** |  | | | | | |  |
| South Africa | 142 | 19 | 1 | 36 | 86 | 34.5 [23.4-47.8] | 98.9 [93.8-99.8] |
| Malawi | 334 | 12 | 37 | 20 | 265 | 37.5 [22.9-54.8] | 87.7 [83.6-91.0] |
| Zambia | 351 | 14 | 58 | 38 | 241 | 26.9 [16.8-40.2] | 80.6 [75.7-84.7] |
| Uganda | 246 | 19 | 33 | 25 | 169 | 43.2 [29.7-57.8] | 83.7 [78.0-88.1] |
| Tanzania | 241 | 8 | 12 | 60 | 161 | 11.8 [6.1-21.5] | 93.1 [88.3-96.0] |
| Vietnam | 175 | 8 | 8 | 24 | 135 | 25.0 [13.2-42.1] | 94.4 [89.3-97.1] |
| Thailand | 127 | 2 | 16 | 6 | 103 | 25.0 [7.1-59.1] | 86.6 [79.3-91.6] |

eMRS, extended microbiological reference standard; FN, false negative; FP, false positive; N, number; TN, true negative; true positive.

**Table 6.** **Sensitivity and specificity of Day 1 and Day 2 FujiLAM against the CRS**

| **Day 1** | **N** | **TP** | **FP** | **FN** | **TN** | **Sensitivity % [95%CI]** | **Specificity % [95%CI]** |
| --- | --- | --- | --- | --- | --- | --- | --- |
| All | 1411 | 218 | 125 | 266 | 802 | 45.0 [40.7-49.5] | 86.5 [84.2-88.6] |
| ***CD4*** |  | | | | | | |
| ≤100 | 264 | 100 | 17 | 50 | 97 | 66.7 [58.8-73.7] | 85.1 [77.4-90.5] |
| 101 to ≤200 | 156 | 31 | 11 | 39 | 75 | 44.3 [33.2-55.9] | 87.2 [78.5-92.7] |
| 201 to ≤500 | 439 | 53 | 48 | 90 | 248 | 37.1 [29.6-45.2] | 83.8 [79.2-87.6] |
| >500 | 543 | 32 | 48 | 83 | 380 | 27.8 [20.4-36.6] | 88.8 [85.4-91.4] |
| Unknown | 9 | 2 | 1 | 4 | 2 | 33.3 [9.7-70.0] | 66.7 [20.8-93.8] |
| ***Setting*** |  | | | | | | |
| Inpatient | 526 | 126 | 26 | 111 | 263 | 53.2 [46.8-59.4] | 91.0 [87.1-93.8] |
| Outpatient | 885 | 92 | 99 | 155 | 539 | 37.2 [31.5-43.4] | 84.5 [81.5-87.1] |
| ***Country*** |  | | | | | | |
| South Africa | 137 | 46 | 16 | 23 | 52 | 66.7 [54.9-76.6] | 76.5 [65.1-85.0] |
| Malawi | 280 | 23 | 31 | 30 | 196 | 43.4 [31.0-56.7] | 86.3 [81.3-90.2] |
| Zambia | 303 | 59 | 25 | 76 | 143 | 43.7 [35.6-52.1] | 85.1 [79.0-89.7] |
| Uganda | 202 | 50 | 11 | 51 | 90 | 49.5 [40.0-59.1] | 89.1 [81.5-93.8] |
| Tanzania | 241 | 21 | 20 | 54 | 146 | 28.0 [19.1-39.0] | 88.0 [82.1-92.1] |
| Vietnam | 135 | 11 | 5 | 23 | 96 | 32.4 [19.1-49.2] | 95.0 [88.9-97.9] |
| Thailand | 113 | 8 | 17 | 9 | 79 | 47.1 [26.2-69.0] | 82.3 [73.5-88.6] |
| **Day 2** | **N** | **TP** | **FP** | **FN** | **TN** | **Sensitivity % [95%CI]** | **Specificity % [95%CI]** |
| All | 1404 | 210 | 153 | 271 | 770 | 43.7 [39.3-48.1] | 83.4 [80.9-85.7] |
| ***CD4*** |  | | | | | | |
| ≤100 | 262 | 101 | 26 | 47 | 88 | 68.2 [60.4-75.2] | 77.2 [68.7-83.9] |
| 101 to ≤200 | 156 | 32 | 15 | 38 | 71 | 45.7 [34.6-57.3] | 82.6 [73.2-89.1] |
| 201 to ≤500 | 436 | 46 | 55 | 96 | 239 | 32.4 [25.2-40.5] | 81.3 [76.4-85.3] |
| >500 | 541 | 29 | 56 | 86 | 370 | 25.2 [18.2-33.9] | 86.9 [83.3-89.7] |
| Unknown | 9 | 2 | 1 | 4 | 2 | 33.3 [9.7-70.0] | 66.7 [20.8-93.8] |
| ***Setting*** |  | | | | | | |
| Inpatient | 519 | 123 | 36 | 112 | 248 | 52.3 [46.0-58.6] | 87.3 [83.0-90.7] |
| Outpatient | 885 | 87 | 117 | 159 | 522 | 35.4 [29.7-41.5] | 81.7 [78.5-84.5] |
| ***Country*** |  | | | | | | |
| South Africa | 133 | 46 | 19 | 22 | 46 | 67.6 [55.8-77.6] | 70.8 [58.8-80.4] |
| Malawi | 279 | 28 | 42 | 25 | 184 | 52.8 [39.7-65.6] | 81.4 [75.8-86.0] |
| Zambia | 301 | 52 | 34 | 81 | 134 | 39.1 [31.2-47.6] | 79.8 [73.0-85.1] |
| Uganda | 202 | 49 | 16 | 52 | 85 | 48.5 [39.0-58.1] | 84.2 [75.8-90.0] |
| Tanzania | 241 | 16 | 18 | 59 | 148 | 21.3 [13.6-31.9] | 89.2 [83.5-93.0] |
| Vietnam | 135 | 11 | 2 | 23 | 99 | 32.4 [19.1-49.2] | 98.0 [93.1-99.5] |
| Thailand | 113 | 8 | 22 | 9 | 74 | 47.1 [26.2-69.0] | 77.1 [67.7-84.4] |

CRS, composite reference standard; FN, false negative; FP, false positive; N, number; TN, true negative; true positive.

**Table 7. Sensitivity and specificity of Day 1 and Day 2 AlereLAM against CRS**

| **Day 1** | **N** | **TP** | **FP** | **FN** | **TN** | **Sensitivity % [95%CI]** | **Specificity % [95%CI]** |
| --- | --- | --- | --- | --- | --- | --- | --- |
| All | 1411 | 146 | 44 | 338 | 883 | 30.2 [26.2-34.4] | 95.3 [93.7-96.4] |
| ***CD4*** |  | | | | | | |
| ≤100 | 264 | 74 | 7 | 76 | 107 | 49.3 [41.4-57.2] | 93.9 [87.9-97.0] |
| 101 to ≤200 | 156 | 20 | 7 | 50 | 79 | 28.6 [19.3-40.1] | 91.9 [84.1-96.0] |
| 201 to ≤500 | 438 | 29 | 9 | 114 | 286 | 20.3 [14.5-27.6] | 96.9 [94.3-98.4] |
| >500 | 544 | 22 | 21 | 93 | 408 | 19.1 [13.0-27.3] | 95.1 [92.6-96.8] |
| Unknown | 9 | 1 | 0 | 5 | 3 | 16.7 [3.0-56.4] | 100.0 [43.9-100.0] |
| ***Setting*** |  | | | | | | |
| Inpatient | 526 | 92 | 14 | 145 | 275 | 38.8 [32.8-45.1] | 95.2 [92.0-97.1] |
| Outpatient | 885 | 54 | 30 | 193 | 608 | 21.9 [17.2-27.4] | 95.3 [93.4-96.7] |
| ***Country*** |  | | | | | | |
| South Africa | 137 | 21 | 1 | 48 | 67 | 30.4 [20.8-42.1] | 98.5 [92.1-99.7] |
| Malawi | 280 | 18 | 16 | 35 | 211 | 34.0 [22.7-47.4] | 93.0 [88.9-95.6] |
| Zambia | 303 | 41 | 9 | 94 | 159 | 30.4 [23.2-38.6] | 94.6 [90.1-97.2] |
| Uganda | 202 | 40 | 3 | 61 | 98 | 39.6 [30.6-49.4] | 97.0 [91.6-99.0] |
| Tanzania | 241 | 14 | 7 | 61 | 159 | 18.7 [11.5-28.9] | 95.8 [91.6-97.9] |
| Vietnam | 136 | 7 | 5 | 27 | 97 | 20.6 [10.3-36.8] | 95.1 [89.0-97.9] |
| Thailand | 112 | 5 | 3 | 12 | 92 | 29.4 [13.3-53.1] | 96.8 [91.1-98.9] |
| **Day 2** | **N** | **TP** | **FP** | **FN** | **TN** | **Sensitivity % [95%CI]** | **Specificity % [95%CI]** |
| All | 1403 | 138 | 81 | 343 | 841 | 28.7 [24.8-32.9] | 91.2 [89.2-92.9] |
| ***CD4*** |  | | | | | | |
| ≤100 | 262 | 67 | 8 | 81 | 106 | 45.3 [37.5-53.3] | 93.0 [86.8-96.4] |
| 101 to ≤200 | 154 | 17 | 6 | 53 | 78 | 24.3 [15.8-35.5] | 92.9 [85.3-96.7] |
| 201 to ≤500 | 437 | 32 | 29 | 110 | 266 | 22.5 [16.4-30.1] | 90.2 [86.2-93.1] |
| >500 | 541 | 20 | 37 | 95 | 389 | 17.4 [11.6-25.3] | 91.3 [88.3-93.6] |
| Unknown | 9 | 2 | 1 | 4 | 2 | 33.3 [9.7-70.0] | 66.7 [20.8-93.8] |
| ***Setting*** |  | | | | | | |
| Inpatient | 519 | 87 | 12 | 147 | 273 | 37.2 [31.2-43.5] | 95.8 [92.8-97.6] |
| Outpatient | 884 | 51 | 69 | 196 | 568 | 20.6 [16.1-26.1] | 89.2 [86.5-91.3] |
| ***Country*** |  | | | | | | |
| South Africa | 133 | 21 | 0 | 47 | 65 | 30.9 [21.2-42.6] | 100.0 [94.4-100.0] |
| Malawi | 279 | 16 | 31 | 37 | 195 | 30.2 [19.5-43.5] | 86.3 [81.2-90.2] |
| Zambia | 301 | 46 | 16 | 88 | 151 | 34.3 [26.8-42.7] | 90.4 [85.0-94.0] |
| Uganda | 202 | 34 | 8 | 67 | 93 | 33.7 [25.2-43.3] | 92.1 [85.1-95.9] |
| Tanzania | 241 | 8 | 12 | 67 | 154 | 10.7 [5.5-19.7] | 92.8 [87.8-95.8] |
| Vietnam | 135 | 8 | 6 | 25 | 96 | 24.2 [12.8-41.0] | 94.1 [87.8-97.3] |
| Thailand | 112 | 5 | 8 | 12 | 87 | 29.4 [13.3-53.1] | 91.6 [84.2-95.7] |

CRS, composite reference standard; FN, false negative; FP, false positive; N, number; TN, true negative; true positive.

**Table 8.** **Sensitivity and specificity of Day 1 and Day 2 FujiLAM against the MRS**

| **Day 1** | **N** | **TP** | **FP** | **FN** | **TN** | **Sensitivity % [95%CI]** | **Specificity % [95%CI]** |
| --- | --- | --- | --- | --- | --- | --- | --- |
| All | 1622 | 156 | 197 | 131 | 1138 | 54.4 [48.6-60.0] | 85.2 [83.2-87.0] |
| ***CD4*** |  | | | | | | |
| ≤100 | 326 | 79 | 44 | 17 | 186 | 82.3 [73.5-88.6] | 80.9 [75.3-85.4] |
| 101 to ≤200 | 184 | 25 | 18 | 15 | 126 | 62.5 [47.0-75.8] | 87.5 [81.1-91.9] |
| 201 to ≤500 | 507 | 35 | 70 | 43 | 359 | 44.9 [34.3-55.9] | 83.7 [79.9-86.9] |
| >500 | 590 | 15 | 64 | 54 | 457 | 21.7 [13.6-32.8] | 87.7 [84.6-90.3] |
| Unknown | 15 | 2 | 1 | 2 | 10 | 50.0 [15.0-85.0] | 90.9 [62.3-98.4] |
| ***Setting*** |  | | | | | | |
| Inpatient | 671 | 96 | 63 | 41 | 471 | 70.1 [61.9-77.1] | 88.2 [85.2-90.7] |
| Outpatient | 951 | 60 | 134 | 90 | 667 | 40.0 [32.5-48.0] | 83.3 [80.5-85.7] |
| ***Country*** |  | | | | | | |
| South Africa | 138 | 37 | 22 | 12 | 67 | 75.5 [61.9-85.4] | 75.3 [65.4-83.1] |
| Malawi | 334 | 20 | 36 | 12 | 266 | 62.5 [45.2-77.1] | 88.1 [83.9-91.3] |
| Zambia | 358 | 33 | 56 | 20 | 249 | 62.3 [48.8-74.1] | 81.6 [76.9-85.6] |
| Uganda | 246 | 32 | 36 | 12 | 166 | 72.7 [58.1-83.7] | 82.2 [76.3-86.8] |
| Tanzania | 242 | 18 | 23 | 50 | 151 | 26.5 [17.4-38.0] | 86.8 [81.0-91.0] |
| Vietnam | 176 | 11 | 5 | 22 | 138 | 33.3 [19.8-50.4] | 96.5 [92.1-98.5] |
| Thailand | 128 | 5 | 19 | 3 | 101 | 62.5 [30.6-86.3] | 84.2 [76.6-89.6] |
| **Day 2** | **N** | **TP** | **FP** | **FN** | **TN** | **Sensitivity % [95%CI]** | **Specificity % [95%CI]** |
| All | 1609 | 145 | 241 | 140 | 1083 | 50.9 [45.1-56.6] | 81.8 [79.6-83.8] |
| ***CD4*** |  | | | | | | |
| ≤100 | 322 | 76 | 58 | 18 | 170 | 80.9 [71.8-87.5] | 74.6 [68.5-79.8] |
| 101 to ≤200 | 185 | 25 | 28 | 15 | 117 | 62.5 [47.0-75.8] | 80.7 [73.5-86.3] |
| 201 to ≤500 | 503 | 29 | 80 | 49 | 345 | 37.2 [27.3-48.3] | 81.2 [77.2-84.6] |
| >500 | 587 | 13 | 74 | 56 | 444 | 18.8 [11.3-29.6] | 85.7 [82.4-88.5] |
| Unknown | 12 | 2 | 1 | 2 | 7 | 50.0 [15.0-85.0] | 87.5 [52.9-97.8] |
| ***Setting*** |  | | | | | | |
| Inpatient | 665 | 88 | 85 | 47 | 445 | 65.2 [56.8-72.7] | 84.0 [80.6-86.8] |
| Outpatient | 944 | 57 | 156 | 93 | 638 | 38.0 [30.6-46.0] | 80.4 [77.4-83.0] |
| ***Country*** |  | | | | | | |
| South Africa | 136 | 37 | 31 | 11 | 57 | 77.1 [63.5-86.7] | 64.8 [54.4-73.9] |
| Malawi | 333 | 21 | 58 | 11 | 243 | 65.6 [48.3-79.6] | 80.7 [75.9-84.8] |
| Zambia | 351 | 29 | 62 | 23 | 237 | 55.8 [42.3-68.4] | 79.3 [74.3-83.5] |
| Uganda | 246 | 28 | 44 | 16 | 158 | 63.6 [48.9-76.2] | 78.2 [72.0-83.4] |
| Tanzania | 241 | 14 | 20 | 54 | 153 | 20.6 [12.7-31.6] | 88.4 [82.8-92.4] |
| Vietnam | 175 | 11 | 2 | 22 | 140 | 33.3 [19.8-50.4] | 98.6 [95.0-99.6] |
| Thailand | 127 | 5 | 24 | 3 | 95 | 62.5 [30.6-86.3] | 79.8 [71.7-86.1] |

MRS, microbiological reference standard; FN, false negative; FP, false positive; N, number; TN, true negative; true positive.

**Table 9.** **Sensitivity and specificity of Day 1 and Day 2 AlereLAM against the MRS**

| **Day 1** | **N** | **TP** | **FP** | **FN** | **TN** | **Sensitivity [95%CI]** | **Specificity [95%CI]** |
| --- | --- | --- | --- | --- | --- | --- | --- |
| All | 1623 | 87 | 124 | 200 | 1212 | 30.3 [25.3-35.9] | 90.7 [89.0-92.2] |
| ***CD4*** |  | | | | | |  |
| ≤100 | 327 | 53 | 36 | 43 | 195 | 55.2 [45.2-64.8] | 84.4 [79.2-88.5] |
| 101 to ≤200 | 184 | 14 | 15 | 26 | 129 | 35.0 [22.1-50.5] | 89.6 [83.5-93.6] |
| 201 to ≤500 | 506 | 13 | 33 | 65 | 395 | 16.7 [10.0-26.5] | 92.3 [89.4-94.5] |
| >500 | 591 | 6 | 39 | 63 | 483 | 8.7 [4.0-17.7] | 92.5 [90.0-94.5] |
| Unknown | 15 | 1 | 1 | 3 | 10 | 25.0 [4.6-69.9] | 90.9 [62.3-98.4] |
| ***Setting*** |  | | | | | |  |
| Inpatient | 672 | 58 | 66 | 79 | 469 | 42.3 [34.4-50.7] | 87.7 [84.6-90.2] |
| Outpatient | 951 | 29 | 58 | 121 | 743 | 19.3 [13.8-26.4] | 92.8 [90.8-94.4] |
| ***Country*** |  | | | | | |  |
| South Africa | 138 | 15 | 1 | 34 | 88 | 30.6 [19.5-44.5] | 98.9 [93.9-99.8] |
| Malawi | 335 | 14 | 22 | 18 | 281 | 43.8 [28.2-60.7] | 92.7 [89.2-95.2] |
| Zambia | 358 | 12 | 46 | 41 | 259 | 22.6 [13.5-35.5] | 84.9 [80.5-88.5] |
| Uganda | 246 | 23 | 28 | 21 | 174 | 52.3 [37.9-66.2] | 86.1 [80.7-90.2] |
| Tanzania | 242 | 13 | 8 | 55 | 166 | 19.1 [11.5-30.0] | 95.4 [91.2-97.7] |
| Vietnam | 177 | 7 | 10 | 26 | 134 | 21.2 [10.7-37.8] | 93.1 [87.7-96.2] |
| Thailand | 127 | 3 | 9 | 5 | 110 | 37.5 [13.7-69.4] | 92.4 [86.2-96.0] |
| **Day 2** | **N** | **TP** | **FP** | **FN** | **TN** | **Sensitivity [95%CI]** | **Specificity [95%CI]** |
| All | 1610 | 80 | 165 | 204 | 1161 | 28.2 [23.3-33.7] | 87.6 [85.7-89.2] |
| ***CD4*** |  | | | | | |  |
| ≤100 | 323 | 50 | 36 | 44 | 193 | 53.2 [43.2-62.9] | 84.3 [79.0-88.4] |
| 101 to ≤200 | 183 | 11 | 16 | 29 | 127 | 27.5 [16.1-42.8] | 88.8 [82.6-93.0] |
| 201 to ≤500 | 505 | 10 | 56 | 67 | 372 | 13.0 [7.2-22.3] | 86.9 [83.4-89.8] |
| >500 | 587 | 7 | 55 | 62 | 463 | 10.1 [5.0-19.5] | 89.4 [86.4-91.8] |
| Unknown | 12 | 2 | 2 | 2 | 6 | 50.0 [15.0-85.0] | 75.0 [40.9-92.8] |
| ***Setting*** |  | | | | | |  |
| Inpatient | 666 | 56 | 62 | 78 | 470 | 41.8 [33.8-50.3] | 88.3 [85.3-90.8] |
| Outpatient | 944 | 24 | 103 | 126 | 691 | 16.0 [11.0-22.7] | 87.0 [84.5-89.2] |
| ***Country*** |  | | | | | |  |
| South Africa | 136 | 17 | 1 | 31 | 87 | 35.4 [23.4-49.6] | 98.9 [93.8-99.8] |
| Malawi | 334 | 12 | 37 | 20 | 265 | 37.5 [22.9-54.8] | 87.7 [83.6-91.0] |
| Zambia | 351 | 14 | 58 | 38 | 241 | 26.9 [16.8-40.2] | 80.6 [75.7-84.7] |
| Uganda | 246 | 19 | 33 | 25 | 169 | 43.2 [29.7-57.8] | 83.7 [78.0-88.1] |
| Tanzania | 241 | 8 | 12 | 60 | 161 | 11.8 [6.1-21.5] | 93.1 [88.3-96.0] |
| Vietnam | 175 | 8 | 8 | 24 | 135 | 25.0 [13.2-42.1] | 94.4 [89.3-97.1] |
| Thailand | 127 | 2 | 16 | 6 | 103 | 25.0 [7.1-59.1] | 86.6 [79.3-91.6] |

MRS, microbiological reference standard; FN, false negative; FP, false positive; N, number; TN, true negative; true positive.

**Table 10. Additional microbiological non-study tests contributing to eMRS**

|  | **South Africa** | **Malawi** | **Zambia** | **Uganda** | **Tanzania** | **Viet Nam** | **Thailand** |
| --- | --- | --- | --- | --- | --- | --- | --- |
| **Additional mycobacterial culture and/or Xpert Ultra from additional samples performed based on routine clinical indication and considered for the eMRS classification** | 112/148 | 0/336 | 13/358 | 0/246 | 0/242 | 3/177 | 1/130 |

eMRS, extended microbiological reference standard.

**Table 11. Summary statistics for the generalized linear mixed model fit on the mismatch ratio between FujiLAM and the reference of the full dataset**

| **Country** | **Chisq** | **Df** | **Pr (>Chisq)** | **Adjusted P** |
| --- | --- | --- | --- | --- |
| ***Age*** | 0·201 | 1 | 0·654 | 0·883 |
| ***Sex*** | 0·91 | 1 | 0·34 | 0·612 |
| ***Country*** | 38·807 | 6 | 7·81e-07 | 3·51e-06 |
| ***Lot*** | 51·4 | 5 | 7·16e-10 | 6·44e-09 |
| ***Visit day*** | 9·095 | 1 | 0·003 | 0·008 |
| ***logCD4*** | 0·101 | 1 | 0·75 | 0·883 |
| ***Urine color*** | 0·882 | 4 | 0·927 | 0·927 |
| ***Urine turbidity*** | 1·73 | 4 | 0·785 | 0·883 |
| ***Setting*** | 2·655 | 1 | 0·103 | 0·232 |

Chisq, chi-squared test; Df, degrees of freedom; Pr, probability.

**Table 12. Detailed results of the generalized linear mixed model fit on the mismatch ratio between FujiLAM and the reference on the full dataset**

|  |  | **Estimate** | **Std. Error** | **z value** | **Pr(>\|z\|)** | **OR** | **2·5 %** | **97·5 %** |
| --- | --- | --- | --- | --- | --- | --- | --- | --- |
| (Intercept) |  | 0·031 | 0·822 | 0·037 | 0·97 | 1·031 | 0·206 | 5·163 |
| ***Age*** |  | 0·005 | 0·01 | 0·449 | 0·654 | 1·005 | 0·984 | 1·025 |
| ***Sex*** | *Male* | 0·229 | 0·24 | 0·954 | 0·34 | 1·258 | 0·785 | 2·015 |
| ***Country*** | *Malawi* | 2·05 | 0·491 | 4·174 | 2·99e-05 | 7·765 | 2·966 | 20·33 |
|  | *Zambia* | 0·819 | 0·492 | 1·665 | 0·096 | 2·267 | 0·865 | 5·945 |
|  | *Uganda* | 0·497 | 0·58 | 0·857 | 0·391 | 1·644 | 0·527 | 5·129 |
|  | *Tanzania* | -0·786 | 0·592 | -1·327 | 0·184 | 0·455 | 0·143 | 1·455 |
|  | *Vietnam* | -0·284 | 0·628 | -0·453 | 0·651 | 0·753 | 0·22 | 2·574 |
|  | *Thailand* | 0·329 | 0·656 | 0·501 | 0·616 | 1·389 | 0·384 | 5·023 |
| ***Lot number*** | *19002* | 1·126 | 0·568 | 1·983 | 0·047 | 3·084 | 1·013 | 9·389 |
|  | *19003* | 0·452 | 0·377 | 1·2 | 0·23 | 1·571 | 0·751 | 3·289 |
|  | *20002* | 3·765 | 1·003 | 3·755 | 1·73e-04 | 43·168 | 6·049 | 308·071 |
|  | *20003* | 2·793 | 0·431 | 6·481 | 9·13e-11 | 16·324 | 7·015 | 37·987 |
|  | *20004* | 1·75 | 0·492 | 3·557 | 3·74e-04 | 5·752 | 2·194 | 15·081 |
| ***Visit day*** | *Day 2* | -0·411 | 0·136 | -3·016 | 0·003 | 0·663 | 0·507 | 0·866 |
| ***CD4*** | *logCD4* | 0·073 | 0·229 | 0·319 | 0·75 | 1·076 | 0·686 | 1·686 |
| ***Urine Color*** | *Clear* | -0·012 | 0·449 | -0·027 | 0·978 | 0·988 | 0·41 | 2·381 |
|  | *Dark yellow* | 0·077 | 0·43 | 0·18 | 0·857 | 1·08 | 0·465 | 2·507 |
|  | *Light yellow* | -0·118 | 0·418 | -0·283 | 0·777 | 0·889 | 0·392 | 2·014 |
|  | *Red* | 16·712 | 1036·024 | 0·016 | 0·987 | 1·81e+07 | 0 |  |
| ***Urine Turbidity*** | *Cloudy* | -0·124 | 0·338 | -0·367 | 0·714 | 0·883 | 0·455 | 1·713 |
|  | *Flocculent* | 16·705 | 1348·11 | 0·012 | 0·99 | 1·80e+07 | 0 |  |
|  | *Opaque* | 0·093 | 0·806 | 0·115 | 0·908 | 1·097 | 0·226 | 5·326 |
|  | *Slightly cloudy* | 0·235 | 0·213 | 1·104 | 0·27 | 1·265 | 0·833 | 1·921 |
| ***Setting*** | *Outpatient* | -0·489 | 0·3 | -1·629 | 0·103 | 0·613 | 0·341 | 1·104 |

Std., standard; OR, odds ratio.

**Table 13. Summary statistics for the generalized linear mixed model fit on the mismatch ratio between FujiLAM and the reference on eMRS positives**

|  | **Chisq** | **Df** | **Pr (>Chisq)** | **Adjusted P** |
| --- | --- | --- | --- | --- |
| ***Age*** | 0·132 | 1 | 0·716 | 0·.887 |
| ***Sex*** | 0·02 | 1 | 0·887 | 0·887 |
| ***Country*** | 4·143 | 6 | 0·657 | 0·887 |
| ***Lot*** | 5·696 | 5 | 0·337 | 0·887 |
| ***Visit day*** | 1·156 | 1 | 0·282 | 0·887 |
| ***logCD4*** | 20·73 | 1 | 5·29e-06 | 4·76e-05 |
| ***Urine color*** | 2·474 | 4 | 0·649 | 0·887 |
| ***Urine turbidity*** | 1·261 | 3 | 0·738 | 0·887 |
| ***Setting*** | 0·066 | 1 | 0·797 | 0·887 |

Chisq, chi-squared test; Df, degrees of freedom; Pr, probability.

**Table 14. Detailed results of the generalized linear mixed model fit on the mismatch ratio between FujiLAM and the reference on eMRS positives**

|  |  | **Estimate** | **Std. Error** | **z value** | **Pr(>\|z\|)** | **OR** | **2·5 %** | **97·5 %** |
| --- | --- | --- | --- | --- | --- | --- | --- | --- |
| (Intercept) |  | 9·326 | 2·235 | 4·172 | 3·02e-05 | 1·12e+04 | 140·483 | 8·97e+05 |
| ***Age*** |  | -0·011 | 0·032 | -0·364 | 0·716 | 0·989 | 0·929 | 1·052 |
| ***Sex*** | *Male* | 0·09 | 0·635 | 0·142 | 0·887 | 1·095 | 0·316 | 3·796 |
| ***Country*** | *Malawi* | -0·461 | 1·241 | -0·371 | 0·71 | 0·631 | 0·055 | 7·18 |
|  | *Zambia* | 0·179 | 1·098 | 0·163 | 0·87 | 1·196 | 0·139 | 10·279 |
|  | *Uganda* | 0·435 | 1·379 | 0·315 | 0·752 | 1·545 | 0·103 | 23·072 |
|  | *Tanzania* | -1·425 | 1·329 | -1·072 | 0·283 | 0·24 | 0·018 | 3·252 |
|  | *Vietnam* | -0·929 | 1·324 | -0·702 | 0·483 | 0·395 | 0·029 | 5·287 |
|  | *Thailand* | 2·042 | 2·344 | 0·871 | 0·384 | 7·71 | 0·078 | 762·232 |
| ***Lot number*** | *19002* | -0·801 | 1·472 | -0·544 | 0·586 | 0·449 | 0·025 | 8·044 |
|  | *19003* | -1·995 | 1·084 | -1·84 | 0·066 | 0·136 | 0·016 | 1·138 |
|  | *20002* | -0·211 | 2·626 | -0·08 | 0·936 | 0·81 | 0·005 | 139·12 |
|  | *20003* | -2·041 | 0·99 | -2·063 | 0·039 | 0·13 | 0·019 | 0·903 |
|  | *20004* | -2·104 | 1·203 | -1·749 | 0·08 | 0·122 | 0·012 | 1·29 |
| ***Visit day*** | *Day 2* | -0·365 | 0·339 | -1·075 | 0·282 | 0·694 | 0·357 | 1·35 |
| ***CD4*** | *logCD4* | -3·097 | 0·68 | -4·553 | 5·29e-06 | 0·045 | 0·012 | 0·171 |
| ***Urine Color*** | *Clear* | -0·509 | 1·01 | -0·504 | 0·614 | 0·601 | 0·083 | 4·355 |
|  | *Dark yellow* | 0·475 | 0·894 | 0·531 | 0·595 | 1·608 | 0·279 | 9·284 |
|  | *Light yellow* | -0·245 | 0·852 | -0·288 | 0·774 | 0·783 | 0·147 | 4·155 |
|  | *Red* | 18·855 | 2671·265 | 0·007 | 0·994 | 1·54e+08 | 0 |  |
| ***Urine Turbidity*** | *Cloudy* | 0·05 | 0·809 | 0·061 | 0·951 | 1·051 | 0·215 | 5·133 |
|  | *Opaque* | 0·044 | 1·447 | 0·03 | 0·976 | 1·045 | 0·061 | 17·816 |
|  | *Slightly cloudy* | 0·583 | 0·556 | 1·048 | 0·295 | 1·791 | 0·602 | 5·329 |
| ***Setting*** | *Outpatient* | 0·211 | 0·82 | 0·257 | 0·797 | 1·234 | 0·248 | 6·154 |

Std., standard; OR, odds ratio.

**Table 15. Summary statistics for the generalized linear mixed model fit on the mismatch ratio between FujiLAM and the reference of eMRS negatives**

|  | **Chisq** | **Df** | **Pr (>Chisq)** | **Adjusted P** |
| --- | --- | --- | --- | --- |
| ***Age*** | 0·063 | 1 | 0·801 | 0·996 |
| ***Sex*** | 6·371 | 1 | 0·012 | 0·017 |
| ***Country*** | 35·864 | 6 | 2·93e-06 | 1·32e-05 |
| ***Lot*** | 97·232 | 5 | 2·02e-19 | 1·82e-18 |
| ***Visit day*** | 7·094 | 1 | 0·008 | 0·014 |
| ***logCD4*** | 8·133 | 1 | 0·004 | 0·012 |
| ***Urine color*** | 0·176 | 4 | 0·996 | 0·996 |
| ***Urine turbidity*** | 0·986 | 4 | 0·912 | 0·996 |
| ***Setting*** | 7·792 | 1 | 0·005 | 0·012 |

Chisq, chi-squared test; Df, degrees of freedom; Pr, probability.

**Table 16. Detailed results of the generalized linear mixed model fit on the mismatch ratio between FujiLAM and the reference on eMRS negatives**

|  |  | **Estimate** | **Std. Error** | **z value** | **Pr(>\|z\|)** | **OR** | **2·5 %** | **97·5 %** |
| --- | --- | --- | --- | --- | --- | --- | --- | --- |
| (Intercept) |  | -1·734 | 0·845 | -2·052 | 0·04 | 0·177 | 0·034 | 0·925 |
| ***Age*** |  | 0·002 | 0·01 | 0·252 | 0·801 | 1·002 | 0·983 | 1·022 |
| ***Sex*** | *Male* | 0·583 | 0·231 | 2·524 | 0·012 | 1·792 | 1·139 | 2·818 |
| ***Country*** | *Malawi* | 2·388 | 0·531 | 4·5 | 6·78e-06 | 10·887 | 3·849 | 30·795 |
|  | *Zambia* | 0·956 | 0·558 | 1·714 | 0·087 | 2·602 | 0·872 | 7·766 |
|  | *Uganda* | 0·297 | 0·618 | 0·481 | 0·63 | 1·346 | 0·401 | 4·519 |
|  | *Tanzania* | 0·382 | 0·733 | 0·521 | 0·603 | 1·465 | 0·348 | 6·168 |
|  | *Vietnam* | 0·202 | 0·837 | 0·241 | 0·809 | 1·224 | 0·237 | 6·318 |
|  | *Thailand* | -0·031 | 0·68 | -0·046 | 0·963 | 0·969 | 0·255 | 3·677 |
| ***Lot number*** | *19002* | 1·765 | 0·538 | 3·282 | 0·001 | 5·843 | 2·036 | 16·762 |
|  | *19003* | 0·776 | 0·336 | 2·309 | 0·021 | 2·174 | 1·125 | 4·201 |
|  | *20002* | 3·787 | 1·012 | 3·741 | 1·83e-04 | 44·12 | 6·067 | 320·846 |
|  | *20003* | 4·35 | 0·465 | 9·352 | 8·64e-21 | 77·484 | 31·136 | 192·826 |
|  | *20004* | 2·451 | 0·475 | 5·165 | 2·41e-07 | 11·603 | 4·577 | 29·412 |
| ***Visit day*** | *Day 2* | -0·402 | 0·151 | -2·663 | 0·008 | 0·669 | 0·498 | 0·899 |
| ***CD4*** | *logCD4* | 0·615 | 0·216 | 2·852 | 0·004 | 1·85 | 1·212 | 2·823 |
| ***Urine Color*** | *Clear* | 0·048 | 0·483 | 0·099 | 0·921 | 1·049 | 0·407 | 2·701 |
|  | *Dark yellow* | 0·055 | 0·467 | 0·117 | 0·907 | 1·056 | 0·423 | 2·638 |
|  | *Light yellow* | -0·026 | 0·45 | -0·057 | 0·955 | 0·975 | 0·404 | 2·354 |
|  | *Red* | 14·919 | 882·136 | 0·017 | 0·987 | 3·02e+06 | 0 |  |
| ***Urine Turbidity*** | *Cloudy* | 0·101 | 0·368 | 0·276 | 0·783 | 1·107 | 0·538 | 2·275 |
|  | *Flocculent* | 13·931 | 714·522 | 0·019 | 0·984 | 1·12e+06 | 0 |  |
|  | *Opaque* | 0·417 | 1·025 | 0·407 | 0·684 | 1·518 | 0·203 | 11·327 |
|  | *Slightly cloudy* | 0·21 | 0·226 | 0·928 | 0·353 | 1·234 | 0·792 | 1·923 |
| ***Setting*** | *Outpatient* | -0·868 | 0·311 | -2·791 | 0·005 | 0·42 | 0·228 | 0·772 |

Std., standard; OR, odds ratio.

**Table 17. 111 FujiLAM-positive, eMRS-negative urine samples tested on all six FujiLAM lots and AlereLAM**

| **Lot** | **Positive/All (%)** |
| --- | --- |
| **19001** | 86/111 (77) |
| **19002** | 77/111 (69) |
| **19003** | 78/111 (71) |
| **20002** | 32/111 (29) |
| **20003** | 14/111 (13) |
| **20004** | 14/111 (13) |
| **AlereLAM** | 9/111 (8) |

**Table 18. EclLAM concentration and FujiLAM results from the six lots used in the study using 70 well-characterized urine samples from the FIND specimen bank**

| **Patient TB Category** | **LAM EclLAM (pg/mL)** | **CD4 (cells/µl)** | **FujiLAM 19001** | **FujiLAM 19002** | **FujiLAM 19003** | **FujiLAM 20002** | **FujiLAM 20003** | **FujiLAM 20004** |
| --- | --- | --- | --- | --- | --- | --- | --- | --- |
| S+C+ | 2031.3 | 56 | POS | POS | POS | POS | POS | POS |
| Xp+S-C- | 210.1 | 485 | POS | POS | POS | POS | POS | POS |
| S+C+ | 165 | 540 | POS | POS | POS | POS | POS | POS |
| S-C+ | 76 | 177 | POS | POS | POS | POS | POS | POS |
| S+C+ | 67 | 49 | POS | POS | POS | POS | POS | POS |
| S+C+ | 50.5 | 30 | POS | POS | POS | POS | POS | POS |
| S+C+ | 46.8 | NA | POS | POS | POS | POS | POS | POS |
| S-C+ | 45 | 156 | POS | POS | POS | POS | POS | POS |
| S+C+ | 44.8 | NA | POS | POS | POS | NEG | NEG | POS |
| S-C+ | 41.7 | NA | POS | POS | POS | POS | POS | POS |
| Xp+S-C- | 41.5 | 505 | POS | POS | POS | POS | POS | POS |
| S+C+ | 39.8 | NA | POS | POS | POS | POS | POS | POS |
| S+C+ | 37 | 115 | POS | POS | POS | POS | POS | POS |
| Xp+S-C- | 34.4 | NA | POS | POS | POS | POS | POS | POS |
| S+C+ | 32.5 | NA | POS | POS | POS | NEG | NEG | NEG |
| S+C+ | 31 | NA | POS | POS | NEG | POS | POS | POS |
| S+C+ | 29.6 | 423 | POS | POS | POS | POS | NEG | NEG |
| S+C+ | 29.4 | NA | POS | POS | POS | POS | POS | POS |
| S+C+ | 29 | NA | POS | POS | POS | POS | POS | POS |
| S+C+ | 28.4 | NA | POS | POS | POS | POS | POS | POS |
| S+C+ | 28 | NA | POS | POS | POS | POS | POS | POS |
| S+C+ | 27 | 646 | POS | POS | POS | POS | POS | NEG |
| S+C+ | 26.3 | NA | POS | POS | POS | NEG | NEG | NEG |
| S+C+ | 26 | 27 | POS | POS | POS | POS | POS | POS |
| S+C+ | 25.1 | NA | POS | POS | POS | POS | NEG | NEG |
| S-C+ | 22.5 | NA | POS | NEG | NEG | NEG | NEG | NEG |
| S+C+ | 22.2 | NA | POS | POS | POS | POS | NEG | NEG |
| S+C+ | 20.5 | 246 | POS | POS | POS | NEG | NEG | POS |
| S+C+ | 20 | NA | POS | POS | POS | POS | NEG | POS |
| S+C+ | 19.2 | NA | POS | POS | POS | POS | POS | NEG |
| S-C+ | 19.2 | NA | POS | POS | POS | POS | POS | POS |
| S+C+ | 18.8 | NA | POS | POS | POS | NEG | NEG | NEG |
| S+C+ | 18.6 | NA | POS | POS | POS | POS | NEG | NEG |
| S+C+ | 18 | NA | POS | POS | POS | POS | NEG | POS |
| S+C+ | 17.9 | 278 | POS | POS | POS | POS | POS | POS |
| S+C+ | 17.3 | NA | POS | POS | POS | POS | POS | POS |
| S+C+ | 16.3 | NA | POS | POS | POS | POS | NEG | POS |
| S-C+ | 16.1 | NA | POS | POS | POS | POS | POS | POS |
| S+C+ | 15.3 | NA | POS | POS | NEG | NEG | NEG | NEG |
| S+C+ | 14.3 | NA | POS | POS | POS | POS | NEG | POS |
| S+C+ | 13.5 | NA | POS | POS | POS | POS | NEG | POS |
| S-C+ | 12.6 | NA | POS | POS | POS | POS | NEG | POS |
| S+C+ | 12.5 | NA | POS | POS | POS | POS | NEG | NEG |
| S+C+ | 12.4 | NA | POS | POS | POS | NEG | NEG | NEG |
| S+C+ | 11.8 | NA | POS | POS | POS | POS | NEG | POS |
| S+C+ | <LoD | NA | POS | POS | POS | NEG | NEG | NEG |
| S+C+ | <LoD | NA | POS | POS | POS | NEG | NEG | NEG |
| S+C+ | <LoD | 264 | POS | POS | POS | NEG | NEG | NEG |
| S+C+ | <LoD | NA | POS | POS | POS | POS | POS | POS |
| S+C+ | <LoD | 486 | POS | POS | POS | NEG | NEG | NEG |
| NonTB | <LoD | 602 | NEG | POS | POS | NEG | NEG | NEG |
| NonTB | <LoD | NA | POS | POS | POS | NEG | NEG | NEG |
| NonTB | <LoD | NA | POS | POS | NEG | NEG | NEG | NEG |
| NonTB | <LoD | NA | POS | POS | POS | NEG | NEG | NEG |
| NonTB | <LoD | NA | NEG | NEG | NEG | NEG | NEG | NEG |
| NonTB | <LoD | 735 | NEG | NEG | NEG | NEG | NEG | NEG |
| NonTB | <LoD | NA | POS | NEG | NEG | NEG | NEG | NEG |
| NonTB | <LoD | NA | POS | POS | POS | NEG | NEG | NEG |
| NonTB | <LoD | NA | POS | POS | POS | NEG | NEG | NEG |
| NonTB | <LoD | NA | POS | POS | POS | NEG | NEG | NEG |
| NonTB | <LoD | NA | POS | POS | POS | NEG | NEG | NEG |
| NonTB | <LoD | NA | POS | POS | POS | NEG | NEG | NEG |
| NonTB | <LoD | NA | POS | POS | POS | NEG | NEG | NEG |
| NonTB | <LoD | NA | POS | POS | POS | NEG | NEG | NEG |
| NonTB | <LoD | NA | POS | POS | POS | POS | NEG | NEG |
| NonTB | <LoD | NA | POS | POS | POS | POS | NEG | NEG |
| NonTB | <LoD | 596 | POS | POS | POS | NEG | NEG | NEG |
| NonTB | <LoD | 98 | POS | POS | POS | NEG | NEG | NEG |
| NonTB | <LoD | 703 | POS | POS | NEG | NEG | NEG | NEG |
| NonTB | <LoD | 265 | NEG | POS | NEG | NEG | NEG | NEG |

C, culture; LoD, limit of detection; NEG, negative; NonTB, patients presenting with signs and symptoms suggestive of TB but negative on all available microbiological tests; POS, positive; TB, tuberculosis; S, smear; Xp, Xpert MTB/Rif; S+C+, sputum culture positive and sputum smear microscopy positive; Xp+S-C-, Sputum Xpert MTB/Rif positive, sputum culture and sputum smear microscopy negative.

**Table 19. FujiLAM lots used in previous studies with adult patients**

|  |  |  |  |  | **HIV +** | | **HIV -** | |
| --- | --- | --- | --- | --- | --- | --- | --- | --- |
| **Study**​ | **Countries**​ | **Population** | **Overall sample size** | **Lot** | **Sensitivity %** | **Specificity %** | **Sensitivity %** | **Specificity %** |
| **Broger T. et al. Lancet Infect Dis. 2019 [1]** | South Africa | Adult inpatients, HIV+ | 968 | 98002 | 70 | 91 | NA | NA |
| **Bjerrum S. et al. Open Forum Infect Dis. 2019 [2]** | Gh​ana | Adult in- and outpatients, HIV+ | 532 | 98004 | 74 | 89 | NA | NA |
| **Broger T. et al. PLoS Med. 2020 [3]** | South Africa Ghana  Viet Nam | Adult in- and outpatients, HIV+ | 1595 | 98002 98004 | 70 | 90 | NA | NA |
| **Muyoyeta M. et al. Eur Respir J. 2021 [4]** | Za​mbia | Adult outpatients, HIV+/- | 151 | 98006 | 77 | 89 | 75 | 95 |
| **Comella-Del-Barrio P. et al. J Clin Med. 2021 [5]** | Nigeria | Adult outpatients, HIV+/- | 204 | 20001 | 70 | 93 | 66 | 99 |
| **Broger T. et al. J Clin Invest. 2020 [6]** | Peru  South Africa​ | Adult outpatients, HIV- | 372 | 98006 | NA | NA | 53 | 99 |
| **Huerga H. et al. Lancet Glob Health. 2023 [7]** | Uganda  Kenya  Mozambique  South Africa | Outpatients ≥15 years,  HIV+ | 1106 | 19003  20002  20003  20004 | 60 | 87 | NA | NA |

**Table 20. Evidence to decision tables for FujiLAM lot 19001**

| **Lot 19001** | **Number of results per 1000 patients tested (95% CI)** | | |
| --- | --- | --- | --- |
|  | Prevalence 1% | Prevalence 10% | Prevalence 30% |
| **True positives** | 7 (6 to 8) | 74 (60 to 84) | 222 (180 to 252) |
| **False negatives** | 3 (2 to 4) | 26 (16 to 40) | 78 (48 to 120) |
| **True negatives** | 705 (591 to 836) | 641 (585 to 690) | 499 (455 to 537) |
| **False positives** | 285 (154 to 399) | 259 (210 to 315) | 201 (163 to 245) |

**Table 21. Evidence to decision tables for FujiLAM lot 20003**

| **Lot 20003** | **Number of results per 1000 patients tested (95% CI)** | | |
| --- | --- | --- | --- |
|  | Prevalence 1% | Prevalence 10% | Prevalence 30% |
| **True positives** | 3 (3 to 4) | 33 (25 to 42) | 99 (75 to 126) |
| **False negatives** | 7 (6 to 7) | 67 (58 to 75) | 201 (174 to 225) |
| **True negatives** | 954 (933 to 968) | 868 (848 to 879) | 674 (659 to 684) |
| **False positives** | 36 (22 to 57) | 32 (21 to 52) | 26 (16 to 41) |
