# Supplementary material for: Prospective multicentre accuracy evaluation of the FUJIFILM SILVAMP TB LAM test for the diagnosis of tuberculosis in people living with HIV demonstrates lot-to-lot variability
Source: PLoS One. 2024 May 31;19(5):e0303846. doi: 10.1371/journal.pone.0303846 (PMC11142480; doi:10.1371/journal.pone.0303846)
Supplement: S6 File — (DOCX) [file pone.0303846.s006.docx]

**S6. Supplementary references**

1. Broger T, Sossen B, du Toit E, Kerkhoff AD, Schutz C, Ivanova Reipold E, et al. Novel lipoarabinomannan point-of-care tuberculosis test for people with HIV: a diagnostic accuracy study. Lancet Infect Dis. 2019;19(8):852-61. Epub 2019/06/04. doi: 10.1016/s1473-3099(19)30001-5. PubMed PMID: 31155318; PubMed Central PMCID: PMCPMC6656794.

2. Bjerrum S, Broger T, Székely R, Mitarai S, Opintan JA, Kenu E, et al. Diagnostic Accuracy of a Novel and Rapid Lipoarabinomannan Test for Diagnosing Tuberculosis Among People With Human Immunodeficiency Virus. Open Forum Infect Dis. 2020;7(1):ofz530. Epub 2020/01/25. doi: 10.1093/ofid/ofz530. PubMed PMID: 31976353; PubMed Central PMCID: PMCPMC6966242.

3. Broger T, Nicol MP, Székely R, Bjerrum S, Sossen B, Schutz C, et al. Diagnostic accuracy of a novel tuberculosis point-of-care urine lipoarabinomannan assay for people living with HIV: A meta-analysis of individual in- and outpatient data. PLoS Med. 2020;17(5):e1003113. Epub 2020/05/02. doi: 10.1371/journal.pmed.1003113. PubMed PMID: 32357197.

4. Muyoyeta M, Kerkhoff AD, Chilukutu L, Moreau E, Schumacher SG, Ruhwald M. Diagnostic accuracy of a novel point-of-care urine lipoarabinomannan assay for the detection of tuberculosis among adult outpatients in Zambia: a prospective cross-sectional study. Eur Respir J. 2021;58(5). Epub 2021/05/01. doi: 10.1183/13993003.03999-2020. PubMed PMID: 33926972.

5. Comella-Del-Barrio P, Bimba JS, Adelakun R, Kontogianni K, Molina-Moya B, Osazuwa O, et al. Fujifilm SILVAMP TB-LAM for the Diagnosis of Tuberculosis in Nigerian Adults. J Clin Med. 2021;10(11). Epub 2021/07/03. doi: 10.3390/jcm10112514. PubMed PMID: 34204120; PubMed Central PMCID: PMCPMC8201264.

6. Broger T, Nicol MP, Sigal GB, Gotuzzo E, Zimmer AJ, Surtie S, et al. Diagnostic accuracy of 3 urine lipoarabinomannan tuberculosis assays in HIV-negative outpatients. J Clin Invest. 2020;130(11):5756-64. Epub 2020/07/22. doi: 10.1172/jci140461. PubMed PMID: 32692731; PubMed Central PMCID: PMCPMC7598043.

7. Huerga H, Bastard M, Lubega AV, Akinyi M, Antabak NT, Ohler L, et al. Novel FujiLAM assay to detect tuberculosis in HIV-positive ambulatory patients in four African countries: a diagnostic accuracy study. Lancet Glob Health. 2023;11(1):e126-e35. Epub 2022/12/16. doi: 10.1016/s2214-109x(22)00463-6. PubMed PMID: 36521944; PubMed Central PMCID: PMCPMC9747168.
