# Supplementary material for: Prospective multicentre accuracy evaluation of the FUJIFILM SILVAMP TB LAM test for the diagnosis of tuberculosis in people living with HIV demonstrates lot-to-lot variability
Source: PLoS One. 2024 May 31;19(5):e0303846. doi: 10.1371/journal.pone.0303846 (PMC11142480; doi:10.1371/journal.pone.0303846)
Supplement: S8 File — (DOCX) [file pone.0303846.s008.docx]

**FujiLAM Study Consortium members**

| **First name** | **Middle name** | **Last name** | **Affiliation** |
| --- | --- | --- | --- |
| Emmanuel |  | Moreau | FIND, Geneva, Switzerland |
| Van Anh | Thi | Nguyen | FIND, Hanoi, Viet Nam |
| Mikashmi |  | Kohli | FIND, Geneva, Switzerland |
| Andrea |  | Cavallini | FIND, Geneva, Switzerland |
| Berra |  | Erkosar | FIND, Geneva, Switzerland |
| Derek | T | Armstrong | FIND, Geneva, Switzerland |
| Sergio |  | Carmona | FIND, Geneva, Switzerland |
| Tobias |  | Broger | 1. FIND, Geneva, Switzerland 2. Division of Infectious Disease and Tropical Medicine, Heidelberg University Hospital and Faculty of Medicine, Heidelberg University, Heidelberg, Germany |
| Samuel | G | Schumacher | World Health Organization, Geneva, Switzerland |
| Minyoi |  | Maimbolwa | Centre for Infectious Diseases research in Zambia, Lusaka, Zambia |
| Brian |  | Shuma | Centre for Infectious Diseases research in Zambia, Lusaka, Zambia |
| Apichaya |  | Khlaiphuengsin | HIV-NAT, Thai Red Cross AIDS Research Centre and Excellent center in Tuberculosis, Faculty of Medicine, Chulalongkorn University, Bangkok, Thailand |
| Apicha |  | Mahanontharit | HIV-NAT, Thai Red Cross AIDS Research Centre and Excellent center in Tuberculosis, Faculty of Medicine, Chulalongkorn University, Bangkok, Thailand |
| Trang | Thi Thu | Pham | Hai Phong University of Medicine and Pharmacy, Hai Phong, Viet Nam |
| Hieu | Thi | Nguyen | Viet Tiep Hospital, Hai Phong, Viet Nam |
| Quang | Van | Nguyen | Hai Phong Lung Hospital, Hai Phong, Viet Nam |
